# Iron-Catalyzed *Para*hydrogen Induced Polarization

**DOI:** 10.1021/jacs.3c07735

**Published:** 2023-09-12

**Authors:** Daniel C. Najera, Alison R. Fout

**Affiliations:** † School of Chemical Sciences, University of Illinois at Urbana−Champaign, Urbana, Illinois 61801, United States; ‡ Department of Chemistry, Texas A&M University, College Station, Texas 77840, United States

## Abstract

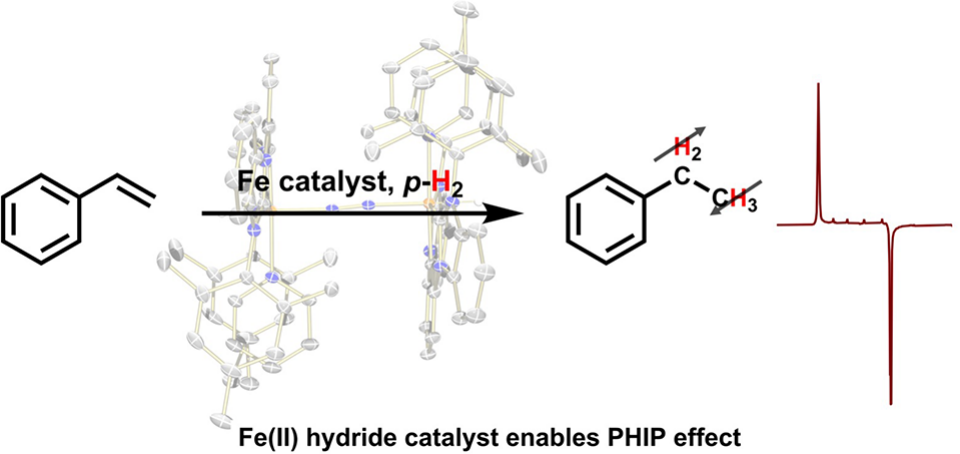

*Para*hydrogen induced polarization (PHIP) can address the low sensitivity problem intrinsic to nuclear magnetic resonance spectroscopy. Using a catalyst capable of reacting with *para*hydrogen and substrate in either a hydrogenative or nonhydrogenative manner can result in signal enhancement of the substrate. This work describes the development of a rare example of an iron catalyst capable of reacting with *para*hydrogen to hyperpolarize olefins. Complexes of the form (^Mes^CCC)Fe(H)(L)(N_2_) (L = Py (Py = pyridine), PMe_3_, PPh_3_) were synthesized from the reaction of the parent complexes (^Mes^CCC)FeMes(L) (Mes = mesityl) with H_2_. The isolated low-spin iron(II) hydride compounds were characterized via multinuclear NMR spectroscopy, infrared spectroscopy, and single crystal X-ray diffraction. (^Mes^CCC)Fe(H)(Py)(N_2_) is competent in the hydrogenation of olefins and demonstrated high activity toward the hydrogenation of monosubstituted terminal olefins. Reactions with *p*-H_2_ resulted in the first PHIP effect mediated by iron which requires diamagnetism throughout the reaction sequence. This work represents the development of a new PHIP catalyst featuring iron, unlocking potential to develop more PHIP catalysts based on first-row transition metals.

## Introduction

*Para*hydrogen (*p*-H_2_), the singlet nuclear spin state of dihydrogen, is an attractive tool to address the problem of low sensitivity intrinsic to nuclear magnetic resonance spectroscopy and imaging techniques through magnetic hyperpolarization via the *para*hydrogen induced polarization (PHIP) effect.^1^ Broadly, PHIP is observed when the incorporation of *p*-H_2_ into a substrate, commonly via (*para*)hydrogenation of an unsaturated carbon–carbon bond mediated by an appropriate catalyst, results in high nuclear spin magnetization and greatly enhanced NMR signals.^2−4^ This is of particular interest to the development of new magnetic resonance imaging (MRI) contrast agents to circumvent the need for gadolinium-based ones that have come under recent scrutiny.^5−8^ Additionally, PHIP methods offer an inexpensive and convenient alternative to the clinically established Dynamic Nuclear Polarization (DNP) that constitutes a minimum expenditure of several million USD and persistent consumption of cryogenics.^9^ Likewise, the application of PHIP in the study of transition metal complexes and their catalytic activity has enabled the detection of short-lived intermediates in low concentrations that would otherwise be undetectable through conventional NMR spectroscopy.^10−14^

Under PHIP conditions, the pure nuclear spin order of *p*-H_2_ generates a non-Boltzmann distribution of nuclear spin states that induces high nuclear spin magnetization in the product with the potential to enhance ^1^H NMR signals by a factor of over 5 orders of magnitude.^2^ The primary conditions require (1) breaking the symmetry of *p*-H_2_ by adding the atoms to magnetically distinct positions, as the nuclear spin singlet is otherwise NMR silent; and (2) the hydrogenation must be faster than the relaxation of the nuclear spin populations. Hyperpolarization is observed as characteristic antiphase signals in the NMR spectrum arising from the selective population of *p*-H_2_-derived spin states in the product.^15^

Since its conception by Bowers and Weitekamp^2^ and subsequent experimental demonstration,^1,3,4^ the landscape of PHIP research has expanded considerably.^16,17^ Of note are the development of non-hydrogenative hyperpolarization (SABRE, signal amplification by reversible exchange),^18−20^ and extension into a broader range of reactivity and biologically relevant substrates^21−23^ from the originally described PASADENA and ALTADENA hydrogenative methods.^24,25^ However, the choice of metal catalyst remains a considerable limiting factor. Systems based on rhodium and iridium have dominated the field since its inception,^1,3,4,17,26−28^ while the fewer examples using palladium^29−32^ and platinum^33^ complement the central role of precious metals in this field. This constitutes a hurdle in the implementation of PHIP in biomedical applications.^34^ Heterogeneous catalytic systems for PHIP^35−38^ and a recent example by Duckett and Weller reported impressive hyperpolarization at room temperature via *para*hydrogenation of gaseous alkenes using a solid-state molecular organometallic (SMOM) catalyst have been reported to address this impediment.^39^ Homogeneous catalysts based on more benign first-row transition metals are an attractive alternative but have remained comparatively underexplored in this area of research, mainly due to the difficulty of meeting the requirements for PHIP in such systems.

**Figure 1:**
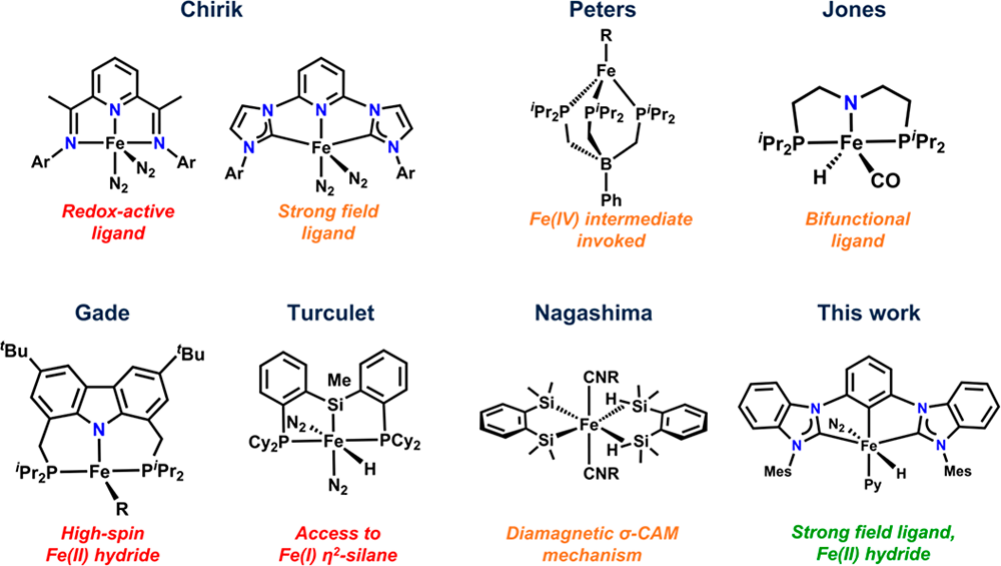
Selected iron catalysts capable of olefin hydrogenation. Listed in red indicates that they are likely not able to be PHIP catalysts and the reason why, orange may be potential PHIP catalysts but have not been tested for PHIP, and green indicates it is a PHIP catalyst.

The high abundance and biocompatibility of iron make it a promising candidate for PHIP catalysis. Indeed, reports on iron hydrogenation catalysts are numerous, and several ligand motifs have been employed in their development.^40^ Work by Chirik,^41−45^ Peters,^46^ Jones,^47^ Gade,^48^ Turculet,^49^ and Nagashima^50,51^ produced representative examples of highly efficient olefin hydrogenation catalysts covering a broad substrate scope and operating through redox-active ligands, metal–ligand cooperativity, oxidative addition/reductive elimination, or σ-CAM (complex assisted metathesis) mechanisms (Figure 1).

Despite great advances in the iron-catalyzed hydrogenation of olefins, the translation to iron-mediated PHIP has not been demonstrated. The reason for this gap was illustrated by Duckett and co-workers^52^ in what is, to the best of our knowledge, the only reported investigation into the interaction between *p*-H_2_ and iron. In that report, a comparative study of the reactivity of M(CO)_3_(dppe) (M = Fe, Ru; dppe = 1,2-bis(diphenylphosphino)ethane) found that the *p*-H_2_ oxidative addition product, M(CO)_2_(dppe)(H)_2_, exhibited polarization of the hydride resonances only in the ruthenium derivative. The lack of signal enhancement of the iron hydride signals was a consequence of a triplet state in the reaction intermediate Fe(CO)_2_(dppe) quenching the *p*-H_2_ spin state. The presence of paramagnetic impurities is known to catalyze the interconversion of *p*-H_2_ back to the equilibrium mixture of *ortho*- and *para*hydrogen (3:1 at 298 K).^12^ Accordingly, factors like structural rearrangement, ligand noninnocence, high-spin electronic configurations, and one-electron oxidation events are detrimental to PHIP and would likely preclude the observation of such an effect on iron (Figure 1).

We recently succeeded in installing iron into a monoanionic bis(NHC) (^Mes^CCC) pincer platform (^Mes^CCC = bis(2,4,6-trimethylphenylbenzimidazol-2-ylidene)phenyl) to furnish a family of Fe(II) complexes (^Mes^CCC)FeMes(L) (**1-L**) (^Mes^CCC = bis(2,4,6-trimethylphenylbenzimidazol-2-ylidene)phenyl; L = pyridine (Py), 3,5-lutidine, PPh_3_, PMe_3_, MeCN, N_2_, CO; Mes = mesityl).^53^ This ligand framework affords efficient, low-spin cobalt hydrogenation catalysts that can utilize *p*-H_2_ to elucidate mechanistic details.^54−57^ We envisioned that similar hydrogenation activity could be accessed by the iron system through activation of H_2_ by **1-L** promoted by protonation of the anionic Mes ligand and extrusion of mesitylene and lead to observation of the PHIP effect. Herein, we report the first example of *para*hydrogen induced polarization mediated by an iron catalyst. The synthesis and characterization of the family of complexes (^Mes^CCC)Fe(H)(L)(N_2_) (**2-L**, L = Py, PMe_3_, PPh_3_) is described, and the catalytic activity toward the hydrogenation of olefins is evaluated. For the hydrogenation catalyst **2-Py**, the combination of a rigid, redox-innocent, and strongly donating CCC ligand framework, compounded with the lability of N_2_ and pyridine ligands, results in a system that circumvents common reactivity deleterious to the PHIP effect.

## Results and Discussion

### Reactivity of 1-Py with H_2_

**Scheme 1:**
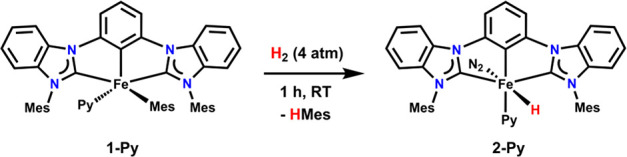
Synthesis of **2-Py**

We began our investigation by exploring the activation of H_2_ by **1-Py** (Py = pyridine) (Scheme 1). The addition of 4 atm of H_2_ to **1-Py** resulted in a gradual color change from dark purple to dark red over the course of 1 h. The ^1^H NMR spectrum of the crude reaction mixture in C_6_D_6_ revealed a new diamagnetic complex and the formation of mesitylene (Figure S9). The alkyl region featured three singlets at 2.24, 2.11, and 1.41 ppm integrating to 6H each assigned to the methyl groups of the flanking mesityl moieties of the CCC ligand framework, consistent with a *C*_*s*_-symmetric complex in solution and markedly shifted from those of the parent compound located at 2.14, 1.45, and 1.16 ppm. A hydridic resonance was identified at −18.84 ppm integrating to 1H, supporting the activation of H_2_ where one H atom undergoes bond formation with the mesityl ligand of **1-Py** and the other is retained as a hydride ligand (Table 1). The resonances in the aryl region integrating to 20H correspond to the aryl backbone of the ^Mes^CCC ligand scaffold and one pyridine ligand. The ATR-IR spectrum of the isolated solid showed an absorbance at 2073 cm^–1^ consistent with an N–N stretch. These spectroscopic features are remarkably similar to those of the previously reported complex (^DIPP^CCC)Fe(H)(Py)(N_2_), which showed a hydride resonance at −18.70 ppm and a N–N stretch at 2081 cm^–1^, supporting the formation of an analogous species with the mesityl derivative of the ligand: (^Mes^CCC)Fe(H)(Py)(N_2_) (**2-Py**). Executing the reaction in Scheme 1 under D_2_ resulted in an identical ^1^H NMR spectrum except for the disappearance of the signal corresponding to the hydride (Figure S9), along with its concomitant appearance in the ^2^H NMR spectrum (Figure S10), showing that the formation of **2-Py** is derived from the activation of H_2_ and extrusion of mesitylene (Scheme 1). These complexes highlight the differing reactivity determined by the choice of flanking aryl groups on the CCC scaffold as **2-Py** could not be synthesized following the zwitterionic metalation strategy used for the ^DIPP^CCC variant^58^ and, conversely, a ^DIPP^CCC analogue of **1-Py** is inaccessible via the reported procedure.^53^

**Table 1:** Selected Spectroscopic Parameters of **2-L**

Complex	δ ^1^H_Fe–H_ (ppm)	δ ^13^C_NHC_ (ppm)	δ ^31^P (ppm)	ν_N–N_ (cm^–1^)
**2-Py**	–18.84	228.09	–	2073
**2-PMe**_**3**_	–9.74	229.98	12.34	2101
**2-PPh**_**3**_	–10.92	228.10	49.13	2096

Although the spectroscopic evidence supports the assigned formulation of **2-Py**, the geometric arrangement of the ligands about the iron center remains dubious. The solid-state structure of the ^DIPP^CCC variant of **2-Py** was not reported and was instead proposed by analogy to the structures determined for phosphine derivatives where the L-type and hydride ligands are in a trans disposition and the bound N_2_ occupies the position trans to the anionic C_Ar_ of the CCC ligand. However, the varying donor strengths and steric imposition of planar pyridine, pyramidal PMe_3_, and bulky PPh_3_ does not discount the possibility of a different geometric arrangement about iron, particularly since pyridine has only been confirmed to bind in a coplanar arrangement with the CCC scaffold and phosphines strictly occupy the apical position in cobalt complexes.^56,59^ This structural trend has also been observed in the iron(II) hydride pincer complexes (^Cy^PSiP^Me^)Fe(H)(N_2_)(L) (PSiP = κ^3^-(2-Cy_2_PC_6_H_4_)_2_SiMe); L = Py, PMe_3_, N_2_) reported by Turculet which show connectivity where pyridine binds to the anionic silyl donor in a *trans* disposition, while PMe_3_ binds trans to the hydride ligand.^49^

**Figure 2:**
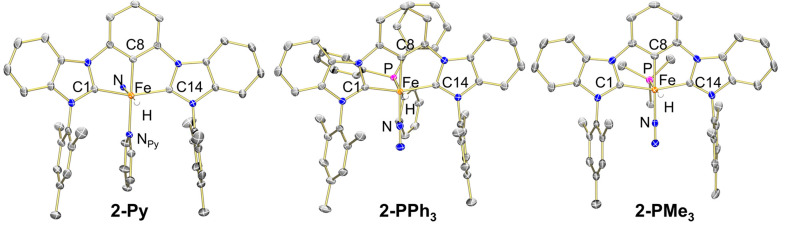
Asymmetric unit of the molecular structure of **2-Py** with 30% probability ellipsoids (left, see SI for full structure); molecular structures of **2-PPh_3_** (center) and **2-PMe_3_** (right) with 50% probability ellipsoids, respectively. Solvent molecules and hydrogen atoms except the hydrides have been omitted for clarity.

Structural characterization of **2-Py** via single-crystal X-ray diffraction revealed a dimeric complex composed of two [(^Mes^CCC)Fe(H)(Py)] fragments bridged by an N_2_ ligand (from crystals grown in a N_2_ filled glovebox) where the hydride ligand occupies the expected position, but the pyridine ligand is positioned trans to the C_Ar_ carbon (Figure 2). The monomeric units are symmetry-related by an inversion centered in the N–N bond, making them magnetically equivalent and indistinguishable from a putative monomeric complex by routine ^1^H or ^13^C NMR spectroscopy. However, it is likely that some monomeric **2-Py** is present in isolated samples due to the weak N_2_ stretch observed in the ATR-IR spectrum. In contrast, the only other reported N_2_-bridged CCC dimer, [(^Mes^CCC)FeMes]_2_(μ-N_2_), remains unambiguously dimeric in solution as determined by the asymmetric ligand environment observed in the ^1^H NMR spectrum.^53^

The ligand arrangement was confirmed to be unique to **2-Py** via the synthesis of the phosphine derivatives (^Mes^CCC)Fe(H)(PR_3_)(N_2_) (R = Me, **2-PMe**_**3**_; and R = Ph, **2-PPh**_**3**_). The addition of H_2_ to **1-PMe**_**3**_ or **1-PPh**_**3**_ resulted in the formation of new diamagnetic complexes with similar ^1^H NMR spectroscopic features to **2-Py**. The appearance of hydride resonances as distinct doublets integrating to 1 H at −9.74 ppm (*J*_P–H_ = 12.7 Hz) and −10.92 ppm (*J*_P–H_ = 22.7 Hz), for PMe_3_ and PPh_3_ respectively, strongly supports the proposed assignment. Comparison of the hydride chemical shifts to their ^DIPP^CCC counterparts (PMe_3_ = −9.67 ppm, *J*_P–H_ = 13 Hz; and PPh_3_ = −11.11 ppm, *J*_P–H_ = 22.5 Hz) support the formation of (^Mes^CCC)Fe(H)(PR_3_)(N_2_) (**2-PMe**_**3**_ and **2-PPh**_**3**_). Additionally, **2-PMe**_**3**_ and **2-PPh**_**3**_ exhibit ^31^P NMR resonances at 12.34 and 49.13 ppm, respectively, comparable to those of the previously reported compounds (11 ppm; and 50 ppm, respectively) that further evidence the proposed formulations. The ATR-IR spectra of **2-PMe**_**3**_ and **2-PPh**_**3**_ show the N_2_ is less activated than in the case of **2-Py**, with strong features at 2101 and 2096 cm^–1^ suggesting the prevalence of the monomeric structure in the solid state (Table 1). Lastly, structural characterization of **2-PR**_**3**_ confirmed the identity of the monomeric product. In contrast with **2-Py**, the phosphine complexes exhibit the expected ligand arrangement with the phosphine and hydride ligand in a *trans* disposition, and the N_2_ opposite the phenylene C_Ar_ atom. Structural parameters for the ligand are similar between the three complexes, negating any change in the electronic structure of the CCC framework between monomer and dimer structures (Table 2).

**Table 2:** Selected Bond Distances and Angles of Complexes **2-L**

	**2-Py**	**2-PPh_3_**	**2-PMe_3_**
Bond Distance (Å)			
Fe–C1	1.9352(14)	1.9228(12)	1.919(3)
Fe–C8	1.8955(14)	1.9017(12)	1.897(3)
Fe–C14	1.9402(14)	1.9332(12)	1.927(3)
Fe–L	2.0609(12)	2.2806(4)	2.2179(12)
Fe–N_2_	1.8733(12)	1.8332(11)	1.840(3)
Fe–H	1.530(19)	1.483(16)	1.50(4)
N–N	1.126(2)	1.1131(6)	1.106(4)
Bond Angles (deg)			
C1–Fe–C14	155.10(6)	157.00(5)	157.64(14)
C8–Fe–N_2_	90.43(6)	166.14(5)	172.95(14)
L–Fe–H	88.3(7)	177.31(8)	173.8(14)
Fe–N–N	174.44(15)	173.75(11)	178.79(16)

### Catalytic Hydrogenation of Olefins

Having established the activation of H_2_, we evaluated the hydrogenation activity of **2-Py**. Initial studies using styrene as a model substrate demonstrated **2-Py** is capable of catalyzing olefin hydrogenation (Table 3). Full conversion to ethylbenzene was observed within 15 min with the catalyst loading of **2-Py** as low as 1 mol % under 4 atm of H_2_ at room temperature, as determined by ^1^H NMR spectroscopy. Dissociation of the pendant pyridine ligand appears necessary for efficient catalysis as the inclusion of 25 mol % of pyridine to the reaction mixture limited conversion of styrene to 68% over the course of 1 h (Table 3, entry 5). The addition of one drop of elemental mercury to the reaction mixture did not hinder this process, supporting, although by no means definitively,^60^ the homogeneous nature of this catalytic system (Table 3, entry 6). Accordingly, **2-PMe**_**3**_ and **2-PPh**_**3**_ only achieved 43% and 45% conversion of styrene after 4 h (Table 3, entries 7 and 8), likely due to the stronger bond formed with phosphorus donors relative to pyridine and further supporting the need to displace the L-type ligand during hydrogenation. However, the different ligand arrangement around the metal center between **2-Py** and **2-PR**_**3**_ does not discount steric effects as the cause for the divergent hydrogenation activity in this family of complexes. Still, displacement of both the L-type ligand and N_2_ is likely necessary in which case the higher activity is still associated with the more labile pyridine. Lastly, while the parent complex **1-Py** was also capable of hydrogenating styrene, a slightly longer reaction time was needed to achieve full conversion, likely to account for the *in situ* formation of **2-Py**.

**Table 3:**
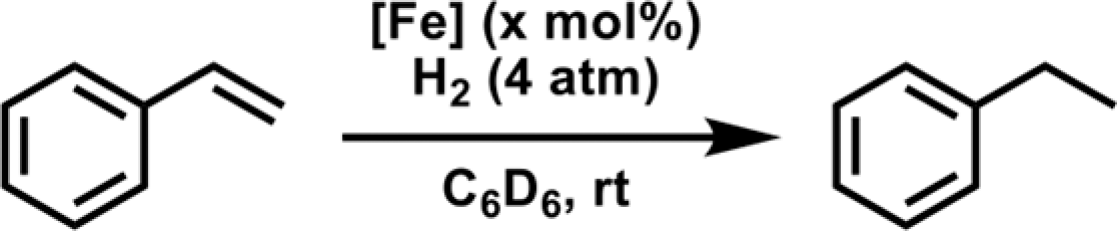
Control Experiments for the Hydrogenation of Styrene

Entry	Catalyst	Catalyst loading^a^	Time	Conversion (%)^b^
1	**2-Py**	2 mol %	1 h	>99
2	**2-Py**	1 mol %	1 h	>99
3	**2-Py**	1 mol %	15 min	>99
4	**2-Py**	0.5 mol %	15 min	70
	**2-Py**	0.1 mol %	15 min	13
5	**2-Py** + Py (25 mol %)	1 mol %	1 h	68
6	**2-Py** + Hg (1 drop)	1 mol %	1 h	>99
7	**2-PPh**_**3**_	2 mol %	4 h	45
8	**2-PMe**_**3**_	2 mol %	4 h	43
9	**1-Py**	2 mol %	30 min	>99%

a All complexes assumed to be monomeric, calculated as mol % of iron in the reaction.

b Conversion determined by ^1^H NMR spectroscopy with mesitylene as an internal standard.

Given the satisfactory hydrogenation activity of **2-Py** and our interest in its potential application toward PHIP, we sought to better understand the capabilities of this system by exploring a representative substrate scope comprised of terminal olefins. A range of terminal alkenes were successfully hydrogenated (Table 4), using the activity of the (^Mes^CCC)Co PHIP catalyst as a benchmark of catalytic performance for this system. The reaction conditions were most amenable to styrene and its electron-rich derivative 4-methoxystyrene, achieving full conversion within 30 min (Table 4, entries 1 and 2). The hydrogenation of the electron-poor 4-fluorostyrene proceeded more gradually to full conversion after 2 h (Table 4, entry 3). Alkyl-substituted olefins, namely 1-octene and 4-vinylcyclohexene (entries 4 and 5), were tolerated as shown by the hydrogenation proceeding to completion in 1 h. Steric bulk in the monosubstituted olefin did not inhibit reactivity, as the more sterically demanding vinyltrimethylsilane was readily hydrogenated to ethyltrimethylsilane in 1 h (Table 4, entry 6). Lastly, ester functionalities were also tolerated with full conversion of methyl-3,3-dimethyl-pentenoate achieved comparatively slowly after 22 h (Table 4, entry 7).

**Table 4:**
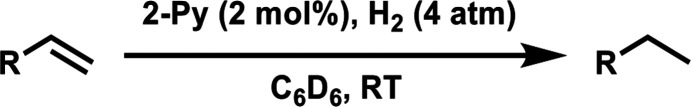

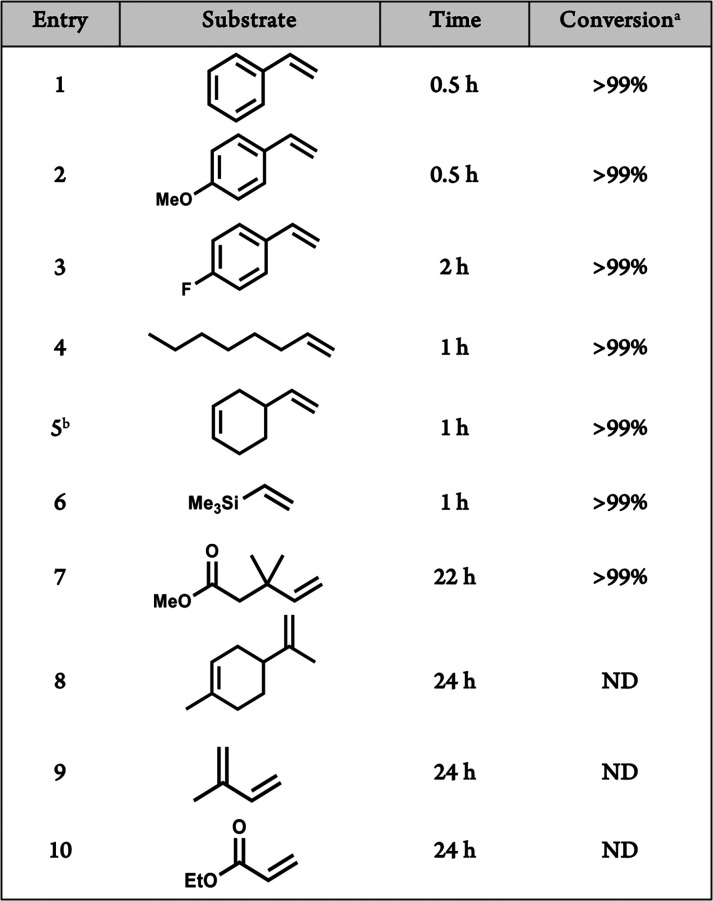
Olefin Hydrogenation by **2-Py**

a Conversion determined by ^1^H NMR spectroscopy with mesitylene as an internal standard.

b Hydrogenation of the terminal olefin exclusively observed.

Some limitations to the hydrogenative capacity of **2-Py** became evident over the course of this investigation. For example, the conversion of 4-vinylcyclohexene to 4-ethylcyclohexane could not be achieved, even after heating the reaction to 80 °C for over 36 h, in stark contrast with our cobalt system which can complete this transformation at 60 °C after 22 h. Likewise, neither the 1,1-disubstituted or trisubstituted olefin moieties of limonene were hydrogenated (Table 4, entry 8), suggesting that this system is mostly limited to monosubstituted alkenes. Isoprene and ethyl acrylate also proved inactive with this system (Table 4, entries 9 and 10).

Overall, the hydrogenation activity of **2-Py** compares favorably with the (^Mes^CCC)Co system, as monosubstituted olefins were hydrogenated in a similar time frame under identical catalyst loadings. While more limited in substrate scope, **2-Py** presents two convenient advantages over the cobalt system: (1) the synthesis of **2-Py** is achieved under mild conditions in a reaction with H_2_ and does not require the use of pyrophoric reductants; and (2) the stability of an 18-electron octahedral Fe(II) complex permits storage of **2-Py** in solution for over 30 days with no noticeable decomposition under an N_2_ atmosphere (Figure S11), whereas (^Mes^CCC)Co(I) fully decomposes over the same period in the solid state at −35 °C. Based, on the comparable activities of the iron and cobalt systems, we surmised that **2-Py** could also serve as a PHIP catalyst.

### *Para*hydrogen Studies

**Figure 3:**
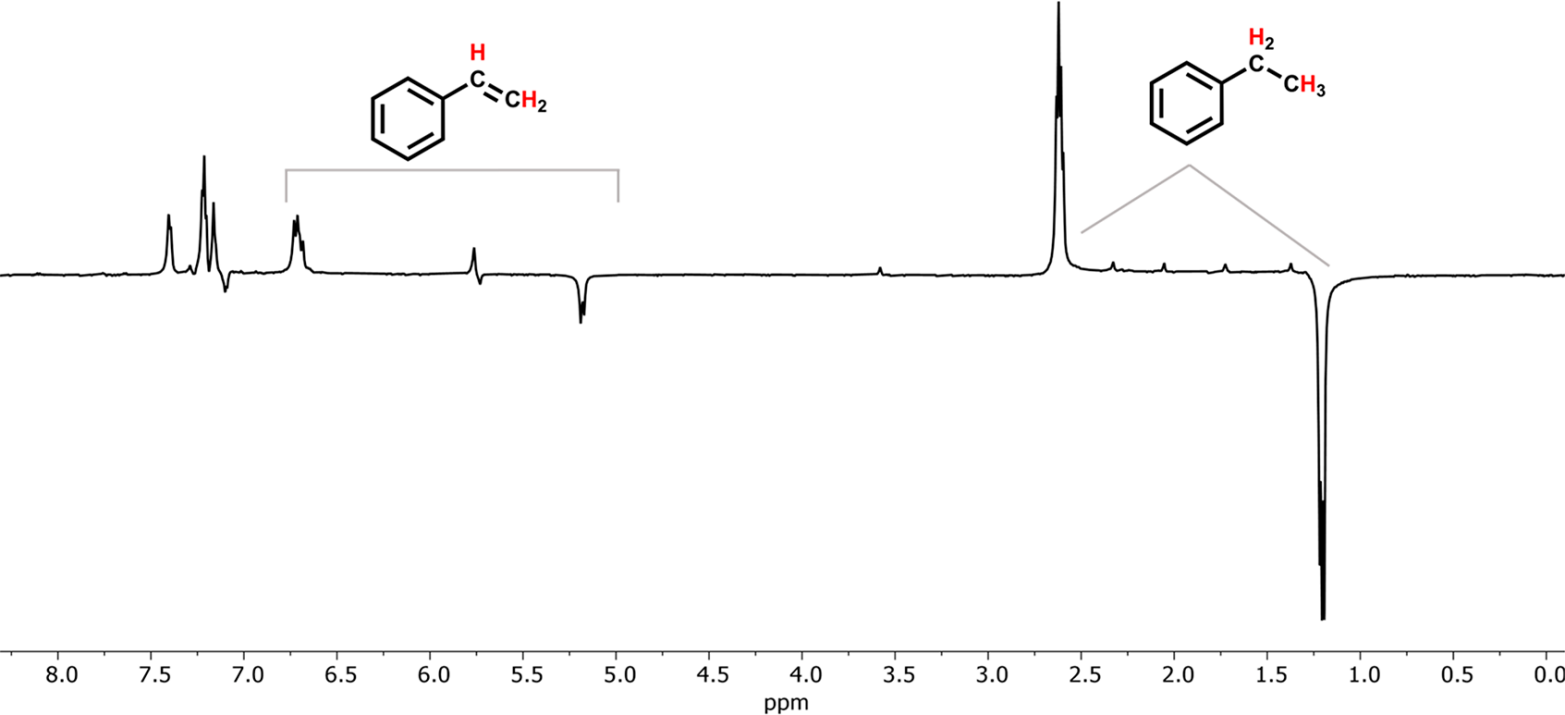
Single transient ^1^H NMR spectrum collected immediately after the introduction of *p*-H_2_ (4 atm) to a solution of styrene and **2-Py** (10 mol %) in THF-*d*_*8*_.

Styrene was chosen as the initial substrate due to the high efficiency at which it could be hydrogenated by **2-Py**, as PHIP requires the addition of H atoms onto the substrate to occur faster than the relaxation of the nuclear spins of the product. Under ALTADENA conditions similar to those used in our cobalt system, a C_6_D_6_ solution of **2-Py** (10 mol %) and styrene was exposed to 4 atm of *p*-H_2_ outside of a 600 MHz NMR spectrometer, followed by immediate collection of an array of one-transient ^1^H NMR spectra upon insertion to the spectrometer. The resulting spectrum exhibited some degree of hyperpolarization, but significant broadening precluded a more informative analysis. Changing the solvent to THF-*d*_*8*_ alleviated this problem and revealed a spectrum featuring two sets of hyperpolarized resonances: a minor set of enhanced signals corresponding to the protons of the substrate and the more prominent set consistent with ethylbenzene (Figure 3). Decay of polarization occurred quickly to full relaxation of the nuclear spins to thermal equilibrium in 6 transients (∼24 s). The methyl and methylene resonances exhibited over 130- and 80-fold signal enhancement, respectively, as determined by the absolute integration of these signals in the hyperpolarized and thermal spectra. Signal enhancements for the hyperpolarized olefin resonances of styrene were within 1 order of magnitude to the thermal values. These signal enhancements are modest compared to those achieved in homogeneous systems based on rhodium^15,61^ and cobalt,^54,57^ which correlates to the poorer hydrogenation efficiency of **2-Py**.

The hyperpolarization of both substrate and product is reminiscent of the results obtained with the (^Mes^CCC)Co(I) system. The appearance of the alkyl protons of ethylbenzene as a pair of antiphase resonances is characteristic of the ALTADENA effect.^4^ Additionally, the hyperpolarization of the intact olefin is akin to the non-hydrogenative SABRE, though mechanistic studies on cobalt have shown that this signal enhancement does not strictly occur by reversible exchange, but through a sequence of insertion and elimination (IE) reactions at cobalt that install individual *p*-H atoms onto the olefinic bonds, termed PHIP-IE.^57^ The collected data suggest that reversible insertion of styrene into an Fe–H bond is involved in the hydrogenation process, although more favorable subsequent steps lead to the prominent signal enhancement of the product resonances.

Substrates that were successfully hydrogenated in Table 3 displayed various degrees of hyperpolarization in the ^1^H NMR spectra when exposed to *p*-H_2_ in the presence of **2-Py**. The substituted styrene derivatives showed similar behavior to the underivatized substrate, with hyperpolarization observed for the olefin and alkyl proton resonances (Figures S25 and S27). The relative hyperpolarization of the olefin protons of 4-fluorostyrene was notably improved with over 40-fold enhancement of these signals compared to the thermal spectrum. This suggests that the reversible insertion of the olefin is favored in more challenging substrates and promotes the PHIP-IE effect.

The olefin protons of 1-octene were hyperpolarized as determined by the decrease in intensity of the associated resonances in the ^1^H NMR spectrum upon relaxation to thermal equilibrium (Figure S29). A third hyperpolarized resonance at about 5.40 ppm was also observed and decayed quickly, which was attributed to a mixture of disubstituted octene resulting from isomerization of the substrate as assessed by comparisons to the reported chemical shifts of these internal alkenes^62^ and commercial samples of *cis*- and *trans*-4-octene. The enhancement of the isomerized alkene resonance in the hyperpolarized spectrum supports olefin insertion into the Fe–H bond, consistent with isomerization occurring during the PHIP-IE process via β-hydride elimination and chain walking of an iron-alkyl intermediate.^63^ Several resonances in the alkyl region of the NMR spectrum exhibited polarization but could not be unambiguously assigned as a result of the similar chemical shifts in the complicated reaction mixture.

The polarized spectrum of 4-vinylcyclohexene further supports β-hydride elimination from a putative iron-alkyl intermediate. The resonance at approximately 0.91 ppm is attributable to the methyl protons of 4-ethylcyclohexene. It appears 1,2-disubstituted olefins can undergo insertion and elimination with **2-Py** and H_2_ as observed in the polarization of a resonance at 5.60 ppm adjacent to that of the internal olefin at 5.63 ppm and assigned to the analogous protons on the hydrogenation product. The reasons underpinning the unsuccessful hydrogenation of the internal olefin despite evidence of insertion into the Fe–H bond are the subject of ongoing investigation.

The most prominent feature of the polarized spectrum of 4-vinylcyclohexene is the inverted resonance at 4.55 ppm consistent with free H_2_ (Figure S31). As mentioned before, the paired nuclear spins of *p*-H_2_ make this molecule NMR silent and should not be observable. Furthermore, the inversion of this signal and eventual decay into conventional, thermally equilibrated, *o*-H_2_ indicates polarization of H_2_. Similar behavior has been previously observed as a byproduct of SABRE^20,64^ and PHIP-IE.^57^ In the latter example, hyperpolarization was proposed to occur as a consequence of regeneration of H_2_ after activation by recombination of hydrides or from polarization of H_2_ via a process akin to HD scrambling from a cobalt(III) dihydrogen-dihydride species. In this case, the viability of an analogous iron(III) dihydrogen-dihydride, (^Mes^CCC)Fe(H)_2_(H_2_), intermediate to promote polarization is unlikely due to the paramagnetic metal center, though a redox-neutral σ-CAM mechanism leading to partner interchange between hydride and dihydrogen ligands in a putative (^Mes^CCC)Fe(H)(H_2_) intermediate cannot be ruled out.^65,66^

**Figure 4:**
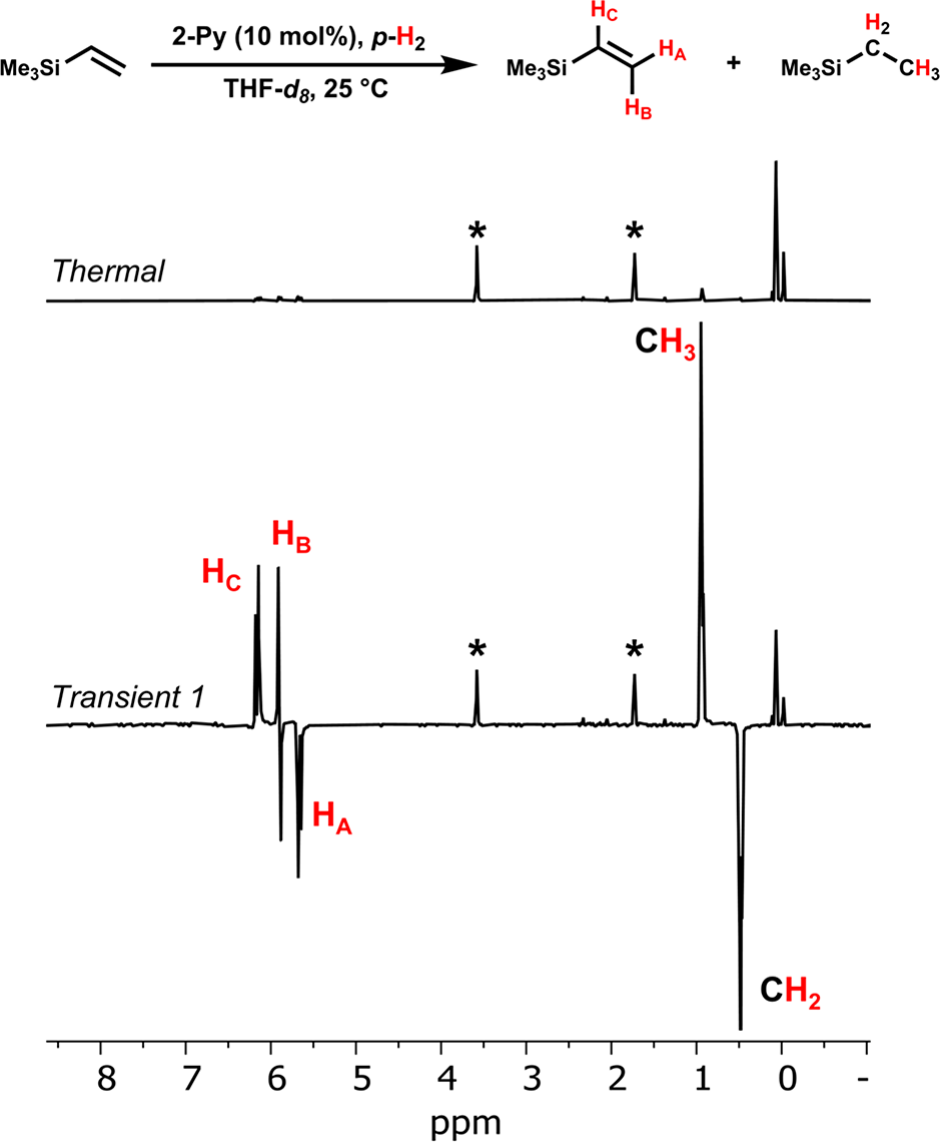
Normalized single transient ^1^H NMR spectra of the reaction of vinyltrimethylsilane with *p*-H_2_ (4 atm) in the presence of **2-Py**. Bottom spectrum was collected immediately following introduction of *p*-H_2_ into the solution; top spectrum was taken after full relaxation of the nuclear spins back to thermal equilibrium. (*) Denotes THF.

Finally, both hydrogenative and non-hydrogenative PHIP effects were best illustrated with vinyltrimethylsilane. The ^1^H NMR spectrum upon addition of *p*-H_2_ showed strong hyperpolarization of the intact olefin and the hydrogenation product (Figure 4). Over 200-fold enhancement over the thermal spectrum was observed for ethyltrimethylsilane. The resonances of the intact substrate exhibited over 30-fold enhancement. Significant consumption of the substrate could be qualitatively assessed by comparison of the trimethylsilyl protons of the product and reactant. These enhancement factors are likely underestimated for the hydrogenative route and overestimated for the non-hydrogenative pathway because of the rapid progression of the reaction. Nevertheless, direct comparison of the absolute integrals of the hyperpolarized resonances and their corresponding trimethylsilyl protons showed over 130-fold enhancement of the methylene bridge signal of the product and a 31-fold enhancement of the methine group in the starting material. Based on these data, it appears this substrate was particularly suited to exhibit the PHIP effect under these conditions relative to the others in this study.

### Mechanistic Considerations

Having explored the reactivity of *p*-H_2_ with a suite of substrates, the specific conditions under which PHIP is observed can be useful in elucidating mechanistic information on the reaction. Chiefly, the iron center remains diamagnetic throughout the reaction, as any paramagnetic state would rapidly catalyze the interconversion to regular H_2_ at the reaction temperature, along with rapid quenching of polarization due to fast relaxation.^52^ This is specially the case here because paramagnetic ferric oxide catalysts are used to generate *p*-H_2_.^67^ The consistent diamagnetic state of iron during catalysis highlights the ability of the strong donor properties of the ^Mes^CCC platform that favors the low-spin state in generated complexes, supplemented by its redox-innocence and rigidity that obviate spin-crossover events detrimental to the PHIP effect.

Hyperpolarization of the structurally intact olefins indicates reversible olefin binding and insertion into the Fe–H bond to generate a putative Fe-alkyl complex. This was confirmed to occur reversibly without the addition of H_2_, as 1-octene exhibited isomerization to internal isomers in the presence of **2-Py** (Figure S22). Olefin isomerization is known to proceed through chain walking via sequential insertion-elimination of the olefin into a metal hydride bond or through allyl-hydride intermediates.^63^ The former is likely operative in this system due to the identity of **2-Py**. This suggests that the initial steps in hydrogenation involve coordination of the olefin followed by migratory insertion into the Fe–H bond.

**Scheme 2:**
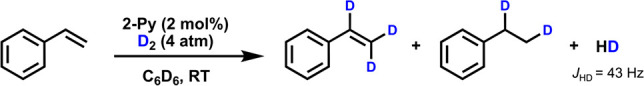
Deuteration of Styrene Catalyzed by **2-Py**

Signal enhancement through PHIP-IE requires incorporation of individual *p*-H_2_ atoms into the substrate without hydrogenation. A second series of insertion–elimination reactions that involve the H_2_ molecule must also be involved in the mechanism. To probe this hypothesis, deuteration experiments were undertaken. Addition of 4 atm of D_2_ under catalytic conditions with styrene and **2-Py** resulted in rapid deuteration of the substrate (Scheme 2). The ^1^H NMR spectrum of the reaction mixture after deuteration showed formation of HD gas, supporting a second insertion–elimination sequence that occurs in the presence of H_2_ (Figure S19). The ^2^H NMR spectrum following addition of D_2_ revealed significant production of deutero-ethylbenzene, as well as the incorporation of deuterium into the olefin moiety of the substrate (Figure S20). A mixture of deuterated substrate and product was also observed with 1-octene, along with concomitant formation of deuterated isomers of octene, consistent with the observed hyperpolarization pattern (Figure S21). This additional non-hydrogenative insertion–elimination route also supports the formation of hyperpolarized H_2_ observed with 4-vinylcyclohexene via analogous steps under which H/D scrambling occurs. Lastly, observation of the characteristic hydride resonance of **2-Py** after hydrogenation suggests this is the resting state of the catalyst.

**Scheme 3:**
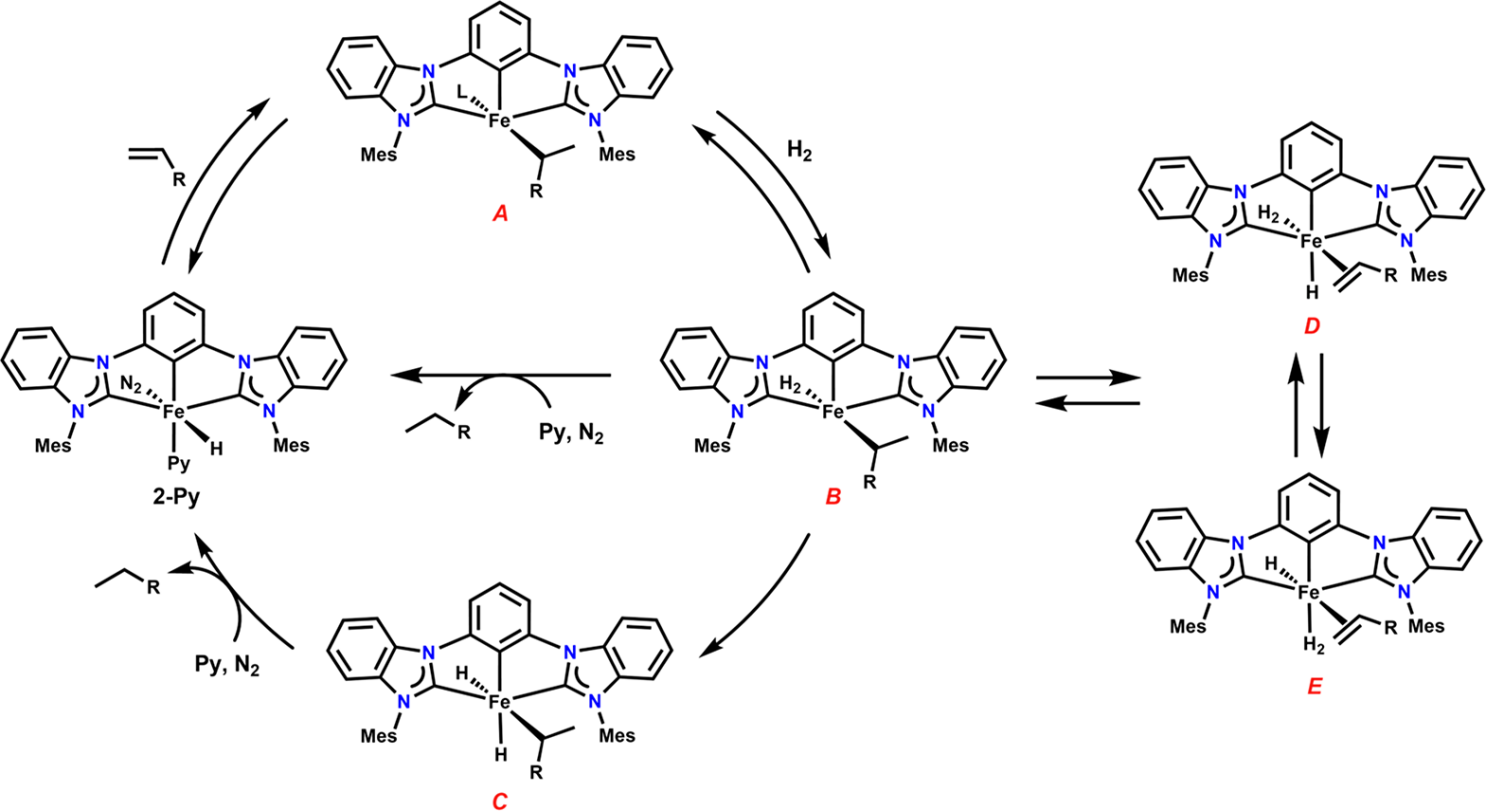
Proposed Mechanism for the Hydrogenation of Olefins by **2-Py**

With the collected data, we propose that the hydrogenation, and *para*hydrogenation, of olefins catalyzed by **2-Py** likely proceeds according to the mechanism in Scheme 3. First, coordination of the olefin through displacement of pyridine or N_2_, or both, leads to migratory insertion to generate an iron-alkyl intermediate **A**. Subsequent coordination of H_2_ generates intermediate **B**, which can undergo β-hydride elimination to regenerate the olefin and the dihydrogen-hydride intermediates **D** and **E**. The hydride and dihydrogen ligands can undergo σ-partner exchange to form HD gas.^65,66^ This exchange process could also explain the incorporation of D or *p*-H atoms into the olefin evidenced in the deuteration of styrene and the observation of the PHIP-IE phenomenon. Lastly, hydrogenation could proceed from **B** through a redox-neutral σ-bond metathesis process, or through the oxidative addition of H_2_ to a putative Fe(IV) intermediate **C**. Both routes were proposed for an iron tris(phosphino)borate complex by Peters (see Figure 1).^46^ Efforts to use *para*hydrogen to distinguish between these pathways by detection of an Fe(IV) intermediate are ongoing. Incorporation of both *p*-H_2_ into the hydrogenation product has long been seen as a requirement for hydrogenative PHIP. In the proposed mechanism, the sequence **B** → **D** → **E** → **B** could potentially install one of the *p*-H_2_ atoms into the substrate, and the following hydrogenation step could install the second atom onto the same substrate. Contrastingly, hyperpolarization of hydrogenation products containing only one *p*-H_2_-derived atom is precedented and termed oneH-PHIP. The possibility of sequential one H-PHIP to result in the observed PHIP effect with **2-Py** cannot be presently discounted.

## Conclusion

Complexes of the form (^Mes^CCC)Fe(H)(L)(N_2_) (**1-L**; L = Py, PMe_3_, PPh_3_) were synthesized from the reaction of the parent complexes (^Mes^CCC)FeMes(L) with H_2_. The isolated low-spin iron(II) hydride compounds were characterized via multinuclear NMR spectroscopy, infrared spectroscopy, and single crystal X-ray diffraction. Notably, **2-Py** is competent in the hydrogenation of olefins comparable to previously reported CCC cobalt systems. A representative substrate scope for this reaction demonstrated high activity toward the hydrogenation of monosubstituted terminal olefins, while a higher degree of substitution was detrimental. Studies with *p*-H_2_ showed, for the first time, *para*hydrogen induced polarization mediated by an iron catalyst. Observation of the PHIP effect implicated a constant diamagnetic state during catalysis, and two hyperpolarization pathways were operative to a different extent in the various substrates. A mechanism was proposed on the basis of the data collected. While signal enhancements were smaller in comparison to state-of-the-art systems, this investigation informs the development of more benign PHIP catalysts for potential biomedical applications.

## Supporting Information Available

Experimental spectra and crystallographic information (PDF)

## References

[ref1] Eisenschmid T. C.; Kirss R. U.; Deutsch P. P.; Hommeltoft S. I.; Eisenberg R.; Bargon J.; Lawler R. G.; Balch A. L. Parahydrogen Induced Polarization in Hydrogenation Reactions. J. Am. Chem. Soc. 1987, 109 (26), 8089–8091. 10.1021/ja00260a026.

[ref2] Bowers C. R.; Weitekamp D. P. Transformation of Symmetrization Order to Nuclear-Spin Magnetization by Chemical Reaction and Nuclear Magnetic Resonance. Phys. Rev. Lett. 1986, 57 (21), 2645–2648. 10.1103/PhysRevLett.57.2645. 10033824

[ref3] Bowers C. R.; Weitekamp D. P. Parahydrogen and Synthesis Allow Dramatically Enhanced Nuclear Alignment. J. Am. Chem. Soc. 1987, 109 (18), 5541–5542. 10.1021/ja00252a049.

[ref4] Pravica M. G.; Weitekamp D. P. Net NMR Alignment by Adiabatic Transport of Parahydrogen Addition Products to High Magnetic Field. Chem. Phys. Lett. 1988, 145 (4), 255–258. 10.1016/0009-2614(88)80002-2.

[ref5] McDonald R. J.; McDonald J. S.; Kallmes D. F.; Jentoft M. E.; Paolini M. A.; Murray D. L.; Williamson E. E.; Eckel L. J. Gadolinium Deposition in Human Brain Tissues after Contrast-Enhanced MR Imaging in Adult Patients without Intracranial Abnormalities. Radiology 2017, 285 (2), 546–554. 10.1148/radiol.2017161595. 28653860

[ref6] Alwasiyah D.; Murphy C.; Jannetto P.; Hogg M.; Beuhler M. C. Urinary Gadolinium Levels After Contrast-Enhanced MRI in Individuals with Normal Renal Function: A Pilot Study. J. Med. Toxicol. 2019, 15 (2), 121–127. 10.1007/s13181-018-0693-1. 30543028 PMC6441052

[ref7] DeBevits J. J.; Munbodh R.; Bageac D.; Wu R.; DiCamillo P. A.; Hu C.; Wang L.; Naismith R. T.; Karimeddini D.; Dhib-Jalbut S.; Redko S.; Cook S. D.; Cadavid D.; Wolansky L. Gray Matter Nucleus Hyperintensity After Monthly Triple-Dose Gadopentetate Dimeglumine With Long-Term Magnetic Resonance Imaging. Invest. Radiol. 2020, 55 (10), 629–635. 10.1097/RLI.0000000000000663. 32898355

[ref8] DeAguero J.; Howard T.; Kusewitt D.; Brearley A.; Ali A.-M.; Degnan J. H.; Jett S.; Watt J.; Escobar G. P.; Dokladny K.; Wagner B. The Onset of Rare Earth Metallosis Begins with Renal Gadolinium-Rich Nanoparticles from Magnetic Resonance Imaging Contrast Agent Exposure. Sci. Rep. 2023, 13 (1), 2025 10.1038/s41598-023-28666-1. 36739294 PMC9899216

[ref9] Stewart N. J.; Matsumoto S. Biomedical Applications of the Dynamic Nuclear Polarization and Parahydrogen Induced Polarization Techniques for Hyperpolarized ^13^C MR Imaging. Magn. Reson. Med. Sci. 2021, 20 (1), 1–17. 10.2463/mrms.rev.2019-0094. 31902907 PMC7952198

[ref10] Kumar A.; Blakemore J. D. On the Use of Aqueous Metal-Aqua pKa Values as a Descriptor of Lewis Acidity. Inorg. Chem. 2021, 60, 1107 10.1021/acs.inorgchem.0c03239. 33405902

[ref11] Godard C.; Duckett S. B.; Polas S.; Tooze R.; Whitwood A. C. Detection of Intermediates in Cobalt-Catalyzed Hydroformylation Using Parahydrogen-Induced Polarization. J. Am. Chem. Soc. 2005, 127 (14), 4994–4995. 10.1021/ja0434533. 15810814

[ref12] Duckett S. B.; Wood N. J. Parahydrogen-Based NMR Methods as a Mechanistic Probe in Inorganic Chemistry. Coord. Chem. Rev. 2008, 252 (21–22), 2278–2291. 10.1016/j.ccr.2008.01.028.

[ref13] Duckett S. B.; Newell C. L.; Eisenberg R. Observation of New Intermediates in Hydrogenation Catalyzed by Wilkinson’s Catalyst, RhCl(PPh_3_)_3_, Using Parahydrogen-Induced Polarization. J. Am. Chem. Soc. 1994, 116 (23), 10548–10556. 10.1021/ja00102a023.

[ref14] Bargon J. Parahydrogen-Induced Polarization: Applications to Detect Intermediates of Catalytic Hydrogenations. In Handbook of Homogeneous Hydrogenation; Wiley-VCH Verlag GmbH: Weinheim, Germany, 2006; pp 313–358. 10.1002/9783527619382.ch12.

[ref15] Green R. A.; Adams R. W.; Duckett S. B.; Mewis R. E.; Williamson D. C.; Green G. G. R. The Theory and Practice of Hyperpolarization in Magnetic Resonance Using Parahydrogen. Prog. Nucl. Magn. Reson. Spectrosc. 2012, 67, 1–48. 10.1016/j.pnmrs.2012.03.001. 23101588

[ref16] Buntkowsky G.; Theiss F.; Lins J.; Miloslavina Y. A.; Wienands L.; Kiryutin A.; Yurkovskaya A. Recent Advances in the Application of Parahydrogen in Catalysis and Biochemistry. RSC Adv. 2022, 12 (20), 12477–12506. 10.1039/D2RA01346K. 35480380 PMC9039419

[ref17] Tickner B. J.; Zhivonitko V. V. Advancing Homogeneous Catalysis for Parahydrogen-Derived Hyperpolarisation and Its NMR Applications. Chem. Sci. 2022, 13 (17), 4670–4696. 10.1039/D2SC00737A. 35655870 PMC9067625

[ref18] Adams R. W.; Duckett S. B.; Green R. A.; Williamson D. C.; Green G. G. R. A Theoretical Basis for Spontaneous Polarization Transfer in Non-Hydrogenative Parahydrogen-Induced Polarization. J. Chem. Phys. 2009, 131 (19), 194505 10.1063/1.3254386. 19929058

[ref19] Adams R. W.; Aguilar J. A.; Atkinson K. D.; Cowley M. J.; Elliott P. I. P.; Duckett S. B.; Green G. G. R.; Khazal I. G.; López-Serrano J.; Williamson D. C. Reversible Interactions with Para-Hydrogen Enhance NMR Sensitivity by Polarization Transfer. Science (80-.). 2009, 323 (5922), 1708–1711. 10.1126/science.1168877. 19325111

[ref20] Barskiy D. A.; Kovtunov K. V.; Koptyug I. V.; He P.; Groome K. A.; Best Q. A.; Shi F.; Goodson B. M.; Shchepin R. V.; Coffey A. M.; Waddell K. W.; Chekmenev E. Y. The Feasibility of Formation and Kinetics of NMR Signal Amplification by Reversible Exchange (SABRE) at High Magnetic Field (9.4 T). J. Am. Chem. Soc. 2014, 136 (9), 3322–3325. 10.1021/ja501052p. 24528143 PMC3985893

[ref21] Glöggler S.; Wagner S.; Bouchard L.-S. Hyperpolarization of Amino Acid Derivatives in Water for Biological Applications. Chem. Sci. 2015, 6 (7), 4261–4266. 10.1039/C5SC00503E. 29218193 PMC5707458

[ref22] Sauer G.; Nasu D.; Tietze D.; Gutmann T.; Englert S.; Avrutina O.; Kolmar H.; Buntkowsky G. Effective PHIP Labeling of Bioactive Peptides Boosts the Intensity of the NMR Signal. Angew. Chem. Int. Ed. 2014, 53 (47), 12941–12945. 10.1002/anie.201404668. 25296746

[ref23] Körner M.; Sauer G.; Heil A.; Nasu D.; Empting M.; Tietze D.; Voigt S.; Weidler H.; Gutmann T.; Avrutina O.; Kolmar H.; Ratajczyk T.; Buntkowsky G. PHIP-Label: Parahydrogen-Induced Polarization in Propargylglycine-Containing Synthetic Oligopeptides. Chem. Commun. 2013, 49 (71), 7839 10.1039/c3cc43978j. 23887355

[ref24] Natterer J.; Bargon J. Parahydrogen Induced Polarization. Prog. Nucl. Magn. Reson. Spectrosc. 1997, 31 (4), 293–315. 10.1016/S0079-6565(97)00007-1.

[ref25] Canet D.; Aroulanda C.; Mutzenhardt P.; Aime S.; Gobetto R.; Reineri F. Para-Hydrogen Enrichment and Hyperpolarization. Concepts Magn. Reson. Part A 2006, 28A (5), 321–330. 10.1002/cmr.a.20065.

[ref26] Eisenschmid T. C.; McDonald J.; Eisenberg R.; Lawler R. G. INEPT in a Chemical Way. Polarization Transfer from Para Hydrogen to Phosphorus-31 by Oxidative Addition and Dipolar Relaxation. J. Am. Chem. Soc. 1989, 111 (18), 7267–7269. 10.1021/ja00200a061.

[ref27] Harthun A.; Selke R.; Bargon J. Proof of a Reversible, Pairwise Hydrogen Transfer during the Homogeneously Rhodium(I)-Catalyzed Hydrogenation of α,β-Unsaturated Carbonic Acid Derivatives with In Situ NMR Spectroscopy and Parahydrogen. Angew. Chem., Int. Ed. 1996, 35 (21), 2505–2507. 10.1002/anie.199625051.

[ref28] Papp G.; Horváth H.; Joó F. A Simple and Efficient Procedure for Rh(I) and Ir(I) complex Catalyzed Para hydrogenation of Alkynes and Alkenes in Aqueous Media Resulting in Strong PHIP Effects. ChemCatChem. 2019, 11 (13), 3000–3003. 10.1002/cctc.201900602.

[ref29] López-Serrano J.; Duckett S. B.; Aiken S.; Almeida Leñero K. Q.; Drent E.; Dunne J. P.; Konya D.; Whitwood A. C. A Para -Hydrogen Investigation of Palladium-Catalyzed Alkyne Hydrogenation. J. Am. Chem. Soc. 2007, 129 (20), 6513–6527. 10.1021/ja070331c. 17469823

[ref30] Harthun A.; Giernoth R.; Elsevier C. J.; Bargon J. Rhodium- and Palladium-Catalysed Proton Exchange in Styrene Detected in Situ by Para-Hydrogen Induced Polarization. Chem. Commun. 1996, (21), 2483–2484. 10.1039/cc9960002483.

[ref31] López-Serrano J.; Duckett S. B.; Dunne J. P.; Godard C.; Whitwood A. C. Palladium Catalysed Alkyne Hydrogenation and Oligomerisation: A Parahydrogen Based NMR Investigation. Dalt. Trans 2008, (32), 4270 10.1039/b804162h. 18682866

[ref32] Guan D.; Jonathan Holmes A.; López-Serrano J.; Duckett S. B. Following Palladium Catalyzed Methoxycarbonylation by Hyperpolarized NMR Spectroscopy: A Parahydrogen Based Investigation. Catal. Sci. Technol. 2017, 7 (10), 2101–2109. 10.1039/C7CY00252A.

[ref33] Permin A. B.; Eisenberg R. One-Hydrogen Polarization in Hydroformylation Promoted by Platinum-Tin and Iridium Carbonyl Complexes: A New Type of Parahydrogen-Induced Effect. J. Am. Chem. Soc. 2002, 124 (42), 12406–12407. 10.1021/ja026698t. 12381170

[ref34] Manoharan A.; Rayner P. J.; Iali W.; Burns M. J.; Perry V. H.; Duckett S. B. Achieving Biocompatible SABRE: An in Vitro Cytotoxicity Study. ChemMedChem. 2018, 13 (4), 352–359. 10.1002/cmdc.201700725. 29232489 PMC5838797

[ref35] Shi F.; Coffey A. M.; Waddell K. W.; Chekmenev E. Y.; Goodson B. M. Heterogeneous Solution NMR Signal Amplification by Reversible Exchange. Angew. Chem. Int. Ed. 2014, 53 (29), 7495–7498. 10.1002/anie.201403135. PMC6284233 24889730

[ref36] Shi F.; Coffey A. M.; Waddell K. W.; Chekmenev E. Y.; Goodson B. M. Nanoscale Catalysts for NMR Signal Enhancement by Reversible Exchange. J. Phys. Chem. C 2015, 119 (13), 7525–7533. 10.1021/acs.jpcc.5b02036. PMC4501382 26185545

[ref37] Kovtunov K. V.; Kovtunova L. M.; Gemeinhardt M. E.; Bukhtiyarov A. V.; Gesiorski J.; Bukhtiyarov V. I.; Chekmenev E. Y.; Koptyug I. V.; Goodson B. M. Heterogeneous Microtesla SABRE Enhancement of ^15^N NMR Signals. Angew. Chem. Int. Ed. 2017, 56 (35), 10433–10437. 10.1002/anie.201705014. PMC5561492 28644918

[ref38] Chekmenev E. Y.; Goodson B. M.; Bukhtiyarov V. I.; Koptyug I. V. Bridging the Gap: From Homogeneous to Heterogeneous Parahydrogen induced Hyperpolarization and Beyond. ChemPhysChem 2021, 22 (8), 710–715. 10.1002/cphc.202001031. 33825286 PMC8357055

[ref39] Gyton M. R.; Royle C. G.; Beaumont S. K.; Duckett S. B.; Weller A. S. Mechanistic Insights into Molecular Crystalline Organometallic Heterogeneous Catalysis through Parahydrogen-Based Nuclear Magnetic Resonance Studies. J. Am. Chem. Soc. 2023, 145 (4), 2619–2629. 10.1021/jacs.2c12642. 36688560 PMC9896567

[ref40] Wei D.; Darcel C. Iron Catalysis in Reduction and Hydrometalation Reactions. Chem. Rev. 2019, 119 (4), 2550–2610. 10.1021/acs.chemrev.8b00372. 30548065

[ref41] Bart S. C.; Lobkovsky E.; Chirik P. J. Preparation and Molecular and Electronic Structures of Iron(0) Dinitrogen and Silane Complexes and Their Application to Catalytic Hydrogenation and Hydrosilation. J. Am. Chem. Soc. 2004, 126 (42), 13794–13807. 10.1021/ja046753t. 15493939

[ref42] Trovitch R. J.; Lobkovsky E.; Bill E.; Chirik P. J. Functional Group Tolerance and Substrate Scope in Bis(Imino)Pyridine Iron Catalyzed Alkene Hydrogenation. Organometallics 2008, 27 (7), 1470–1478. 10.1021/om701091z.

[ref43] Yu R. P.; Darmon J. M.; Hoyt J. M.; Margulieux G. W.; Turner Z. R.; Chirik P. J. High-Activity Iron Catalysts for the Hydrogenation of Hindered, Unfunctionalized Alkenes. ACS Catal. 2012, 2 (8), 1760–1764. 10.1021/cs300358m. 26229734 PMC4517477

[ref44] Chirik P. J. Iron- and Cobalt-Catalyzed Alkene Hydrogenation: Catalysis with Both Redox-Active and Strong Field Ligands. Acc. Chem. Res. 2015, 48 (6), 1687–1695. 10.1021/acs.accounts.5b00134. 26042837

[ref45] Arevalo R.; Chirik P. J. Enabling Two-Electron Pathways with Iron and Cobalt: From Ligand Design to Catalytic Applications. J. Am. Chem. Soc. 2019, 141 (23), 9106–9123. 10.1021/jacs.9b03337. 31084022 PMC6561843

[ref46] Daida E. J.; Peters J. C. Considering Fe^II/IV^ Redox Processes as Mechanistically Relevant to the Catalytic Hydrogenation of Olefins by [PhBP^*i*Pr^_3_]Fe-H x Species. Inorg. Chem. 2004, 43 (23), 7474–7485. 10.1021/ic0488583. 15530098

[ref47] Xu R.; Chakraborty S.; Bellows S. M.; Yuan H.; Cundari T. R.; Jones W. D. Iron-Catalyzed Homogeneous Hydrogenation of Alkenes under Mild Conditions by a Stepwise, Bifunctional Mechanism. ACS Catal. 2016, 6 (3), 2127–2135. 10.1021/acscatal.5b02674.

[ref48] Ott J. C.; Blasius C. K.; Wadepohl H.; Gade L. H. Synthesis, Characterization, and Reactivity of a High-Spin Iron(II) Hydrido Complex Supported by a PNP Pincer Ligand and Its Application as a Homogenous Catalyst for the Hydrogenation of Alkenes. Inorg. Chem. 2018, 57 (6), 3183–3191. 10.1021/acs.inorgchem.7b03227. 29474088

[ref49] Murphy L. J.; Ferguson M. J.; McDonald R.; Lumsden M. D.; Turculet L. Synthesis of Bis(Phosphino)Silyl Pincer-Supported Iron Hydrides for the Catalytic Hydrogenation of Alkenes. Organometallics 2018, 37 (24), 4814–4826. 10.1021/acs.organomet.8b00807.

[ref50] Sunada Y.; Tsutsumi H.; Shigeta K.; Yoshida R.; Hashimoto T.; Nagashima H. Catalyst Design for Iron-Promoted Reductions: An Iron Disilyl-Dicarbonyl Complex Bearing Weakly Coordinating Η_2_-(H-Si) Moieties. Dalt. Trans. 2013, 42 (48), 16687 10.1039/c3dt52598h. 24154529

[ref51] Sunada Y.; Ogushi H.; Yamamoto T.; Uto S.; Sawano M.; Tahara A.; Tanaka H.; Shiota Y.; Yoshizawa K.; Nagashima H. Disilaruthena- and Ferracyclic Complexes Containing Isocyanide Ligands as Effective Catalysts for Hydrogenation of Unfunctionalized Sterically Hindered Alkenes. J. Am. Chem. Soc. 2018, 140 (11), 4119–4134. 10.1021/jacs.8b00812. 29505246

[ref52] Schott D.; Callaghan P.; Dunne J.; Duckett S. B.; Godard C.; Goicoechea J. M.; Harvey J. N.; Lowe J. P.; Mawby R. J.; Müller G.; Perutz R. N.; Poli R.; Whittlesey M. K. The Reaction of M(CO)_3_(Ph_2_PCH_2_CH_2_PPh_2_) (M = Fe, Ru) with Parahydrogen: Probing the Electronic Structure of Reaction Intermediates and the Internal Rearrangement Mechanism for the Dihydride Products. Dalt. Trans. 2004, 3 (20), 3218–3224. 10.1039/B407457B. 15483704

[ref53] Najera D. C.; Peñas-Defrutos M. N.; García-Melchor M.; Fout A. R. γ-Agostic Interactions in (^Mes^CCC)Fe-Mes(L) Complexes. Chem. Commun. 2022, 58 (69), 9626–9629. 10.1039/D2CC03260K. 35959650

[ref54] Tokmic K.; Markus C. R.; Zhu L.; Fout A. R. Well-Defined Cobalt(I) Dihydrogen Catalyst: Experimental Evidence for a Co(I)/Co(III) Redox Process in Olefin Hydrogenation. J. Am. Chem. Soc. 2016, 138 (36), 11907–11913. 10.1021/jacs.6b07066. 27569420

[ref55] Tokmic K.; Fout A. R. Alkyne Semihydrogenation with a Well-Defined Nonclassical Co-H_2_ Catalyst: A H_2_ Spin on Isomerization and E-Selectivity. J. Am. Chem. Soc. 2016, 138 (41), 13700–13705. 10.1021/jacs.6b08128. 27709917

[ref56] Tokmic K.; Jackson B. J.; Salazar A.; Woods T. J.; Fout A. R. Cobalt-Catalyzed and Lewis Acid-Assisted Nitrile Hydrogenation to Primary Amines: A Combined Effort. J. Am. Chem. Soc. 2017, 139 (38), 13554–13561. 10.1021/jacs.7b07368. 28906106

[ref57] Muhammad S. R.; Greer R. B.; Ramirez S. B.; Goodson B. M.; Fout A. R. Cobalt-Catalyzed Hyperpolarization of Structurally Intact Olefins. ACS Catal. 2021, 11 (4), 2011–2020. 10.1021/acscatal.0c03727.

[ref58] Jackson B. J.; Najera D. C.; Matson E. M.; Woods T. J.; Bertke J. A.; Fout A. R. Synthesis and Characterization of (^DIPP^CCC)Fe Complexes: A Zwitterionic Metalation Method and CO_2_ Reactivity. Organometallics 2019, 38 (15), 2943–2952. 10.1021/acs.organomet.9b00271.

[ref59] Ibrahim A. D.; Tokmic K.; Brennan M. R.; Kim D.; Matson E. M.; Nilges M. J.; Bertke J. A.; Fout A. R. Monoanionic Bis(Carbene) Pincer Complexes Featuring Cobalt(I-III) Oxidation States. Dalt. Trans. 2016, 45 (24), 9805–9811. 10.1039/C5DT04723D. 26778113

[ref60] Widegren J. A.; Finke R. G. A Review of the Problem of Distinguishing True Homogeneous Catalysis from Soluble or Other Metal-Particle Heterogeneous Catalysis under Reducing Conditions. J. Mol. Catal. A Chem. 2003, 198 (1–2), 317–341. 10.1016/S1381-1169(02)00728-8.

[ref61] Giernoth R.; Huebler P.; Bargon J. Intermediate Product-Catalyst Complexes in the Homogeneous Hydrogenation of Styrene Derivatives with Parahydrogen and Cationic Rh^I^ Catalysts. Angew. Chem. Int. Ed. 1998, 37 (18), 2473–2475. 10.1002/(SICI)1521-3773(19981002)37:18<2473::AID-ANIE2473>3.0.CO;2-J. 29711366

[ref62] Wang Y.; Qin C.; Jia X.; Leng X.; Huang Z. An Agostic Iridium Pincer Complex as a Highly Efficient and Selective Catalyst for Monoisomerization of 1-Alkenes to Trans-2-Alkenes. Angew. Chem. Int. Ed. 2017, 56 (6), 1614–1618. 10.1002/anie.201611007. 28042692

[ref63] Biswas S. Mechanistic Understanding of Transition-Metal-Catalyzed Olefin Isomerization: Metal-Hydride Insertion-Elimination vs. π-Allyl Pathways. Comments Inorg. Chem. 2015, 35 (6), 300–330. 10.1080/02603594.2015.1059325.

[ref64] Alam M. S.; Li X.; Brittin D. O.; Islam S.; Deria P.; Chekmenev E. Y.; Goodson B. M. Anomalously Large Antiphase Signals from Hyperpolarized Orthohydrogen Using a MOF-Based SABRE Catalyst. Angew. Chem. Int. Ed. 2023, 62 (8), e202213581 10.1002/anie.202213581. 36526582

[ref65] Perutz R. N.; Sabo Etienne S.; Weller A. S. Metathesis by Partner Interchange in Σ Bond Ligands: Expanding Applications of the σ-CAM Mechanism. Angew. Chem. Int. Ed. 2022, 61 (5), e202111462 10.1002/anie.202111462. PMC9299125 34694734

[ref66] Perutz R. N.; Sabo-Etienne S. The σ-CAM Mechanism: σ Complexes as the Basis of σ-Bond Metathesis at Late-Transition-Metal Centers. Angew. Chem. Int. Ed. 2007, 46 (15), 2578–2592. 10.1002/anie.200603224. 17380532

[ref67] Tom B. A.; Bhasker S.; Miyamoto Y.; Momose T.; McCall B. J. Producing and Quantifying Enriched Para-H_2_. Rev. Sci. Instrum. 2009, 80 (1), 016108 10.1063/1.3072881. 19191469

