# Supplementary material for: Iron-Catalyzed Parahydrogen Induced Polarization
Source: J Am Chem Soc. 2023 Sep 12;145(38):21086–95. doi: 10.1021/jacs.3c07735 (PMC10863066; doi:10.1021/jacs.3c07735)
Supplement: Supplementary file 1 — ja3c07735_si_001.pdf [file ja3c07735_si_001.pdf]

Iron-catalyzed *Para*hydrogen Induced Polarization

Daniel C. Najera[a] *and Alison R. Fout*[b]*

[a] School of Chemical Sciences, University of Illinois at Urbana-Champaign, 600 S. Mathews Ave., Urbana, Illinois 61801, USA.

[b] Department of Chemistry, Texas A&M University, College Station, TX 77840, USA.

.

Supporting Information Table of Contents:

General Considerations. 1

Synthesis of Metal Complexes 2

NMR spectra of Metal Complexes 3

Reaction of 1-Py with D_2: synthesis of (^{Mes}CCC)Fe(D)(Py)(N_2) 7

Stability of 2-Py in solution 8

Olefin hydrogenation with 2-Py 9

NMR Spectra of Hydrogenation Reactions 10

Para-Hydrogen Studies 19

Crystallographic parameters 31

References 32

General Considerations. All manipulations of air- and moisture-sensitive compounds were carried out in the absence of water and dioxygen in an MBraun inert atmosphere glovebox under a dinitrogen atmosphere except where specified otherwise. All glassware was oven dried for a minimum of 8 h and cooled in an evacuated antechamber prior to use in the glovebox. Solvents for sensitive manipulations were dried and deoxygenated on a Glass Contour System (SG Water USA, Nashua, NH) and stored over 4 Å molecular sieves purchased from Strem following a literature procedure prior to use.[1] The complexes (^{Mes}CCC)FeMes(L) (L = pyridine, PMe_3, PPh_3) were prepared according to literature procedure.[2] Benzene-d_6 and THF-d_8 were purchased from Cambridge Isotope Labs and were degassed and stored over 4 Å molecular sieves prior to use. Celite® 545 (J. T. Baker) was dried in a Schlenk flask for 24 h under dynamic vacuum while heating to at least 150˚C prior to use in a glovebox.

NMR Spectra were recorded at room temperature on a Bruker spectrometer equipped with a Prodigy probe and a SampleXpress autosampler operating at 600 MHz (^{1}H), 151 MHz (^{13}C), and 243 MHz (^{31}P) and referenced to the residual solvent resonance (δ in parts per million, and J in Hz). Solid-state infrared spectra were recorded using a PerkinElmer Frontier FT-IR spectrophotometer equipped with a KRS5 thallium bromide/iodide Universal Attenuated Total Reflectance accessory. Elemental analyses were performed at the University of Illinois at Urbana-Champaign School of Chemical Sciences Microanalysis Laboratory in Urbana, IL. Mass Spectrometry analyses were performed at the University of Illinois at Urbana-Champaign Mass Spectrometry Laboratory. X-ray crystallography was performed at the George L. Clark X-ray Facility at UIUC. Single-crystal X-ray diffraction data were collected with the use of multimirror monochromatized Mo Kα radiation (0.71073 Å) at 100 K on a Bruker D8 Venture diffractometer equipped with a Photon 100 detector. Combinations of 0.5° φ and ω scans were used to collect the data. The collection, cell refinement, and integration of intensity data were carried out with the APEX2 software.[3] Multi-scan absorption correction was performed using SADABS.[4] The structures were solved with XT[5] and refined with the full-matrix least-squares SHELXL[6] program within the Olex2[7] refinement GUI. All structures were submitted to the Cambridge Structural Database.

Synthesis of Metal Complexes

Synthesis of (^{Mes}CCC)Fe(H)(Py)(N_2) (2-Py). A 15 mL Schlenk flask equipped with a stir bar was charged with (^{Mes}CCC)FeMes(Py) (0.050 g, 0.063 mmol, 1.0 equiv.) and THF (2 mL). The flask was taken out of the glovebox and subjected to two freeze-pump-thaw cycles prior to addition of 1 atm of H_2 at 77 K. The flask was reintroduced to the glovebox and set to stir for 4 h, exhibiting a color change from dark purple to dark red. Afterwards, the solution was filtered over Celite. Volatiles were removed under reduced pressure and the solid residue was washed with cold HMDSO (2 x 2 mL) followed by extraction in hexanes. After removal of volatiles under reduced pressure, the product was obtained as a dark red powder in moderate yield (0.033 g, 0.046 mmol, 74%). Crystals suitable for X-ray diffraction were grown from a concentrated hexanes solution of the product at -35 °C. Anal. Calcd. for $C_{43}H_{39}FeN_7$: C, 72.78; H, 5.54; N, 13.82. Found C, 72.97; H, 5.89; N, 13.69. NMR data (C_6D_6, 25 °C): ^{1}H δ = 7.92 (multiplet, 4H), 7.79 (d, *J* = 7.6 Hz, 2H), 7.54 (t, *J* = 7.6 Hz, 1H), 7.08 (t, *J* = 7.8 Hz, 2H), 6.92 (t, *J* = 7.6 Hz, 2H), 6.87 (s, 4H), 6.64 (d, *J* = 7.2 Hz, 2H), 6.28 (t, *J* = 7.5 Hz, 1H), 5.77 (t, *J* = 6.7 Hz, 2H), 2.24 (s, 6H), 2.11 (s, 6H), 1.41 (s, 6H), -18.83 (s, 1H). ^{13}C{^{1}H} δ = 228.09, 190.32, 190.23, 154.08, 149.01, 138.91, 138.51, 138.47, 136.12, 134.63, 132.84, 132.26, 130.27, 122.71, 122.01, 121.60, 120.59, 109.82, 108.52, 106.46, 21.18, 18.49, 17.15. ATR-IR = 2072 cm^{-1} (N_2).

Synthesis of (^{Mes}CCC)Fe(H)(PMe_3)(N_2) (2-PMe_3). A 15 mL Schlenk flask equipped with a stir bar was charged with (^{Mes}CCC)FeMes(PMe_3) (0.040 g, 0.050 mmol, 1.0 equiv.) and THF (4 mL). The flask was taken out of the glovebox and subjected to two freeze-pump-thaw cycles prior to addition of 1 atm of H_2 at 77 K. The flask was reintroduced to the glovebox and set to stir overnight to ensure full conversion. After stirring, the solution was filtered over Celite and volatiles removed under reduced pressure. The solid residue was washed with HMDSO (3 x 3 mL) and hexanes (2 x 1 mL), and lyophilized from benzene to give a bright orange powder in good yield (0.032 g, 0.045 mmol, 90%). Crystals suitable for X-ray diffraction were grown from a 1:1 solution of diethyl ether and HMDSO. Anal. Calcd. for $C_{41}H_{43}FeN_6P$: C, 69.69; H, 6.13; N, 11.89. Found C, 70.26; H, 5.87; N, 11.43. NMR data (C_6D_6, 25 °C): ^{1}H δ = 7.88 (d, *J* = 8.1 Hz, 2H), 7.65 (d, *J* = 7.5 Hz, 2H), 7.42 (t, *J* = 7.7 Hz, 1H), 7.10 (t, *J* = 7.6 Hz, 2H), 6.96 (t, *J* = 7.6 Hz, 2H), 6.86 (s, 2H), 6.72 (s, 2H), 6.66 (d, *J* = 7.6 Hz, 2H), 2.24 (s, 6H), 2.07 (s, 6H), 2.03 (s, 6H), 0.48 (d, *J* = 6.8 Hz, 9H), -9.52 (d, *J* = 12.7 Hz, 1H). ^{13}C{^{1}H} δ = 229.94 (d, *J* = 14.0 Hz), 185.56 (d, *J* = 18.5 Hz), 147.13, 138.78, 138.52, 138.45, 135.28, 134.33, 132.47, 130.52, 128.88, 121.99, 121.64, 120.13, 109.68, 108.54, 105.94, 21.04, 18.87, 18.70, 15.59 (d, *J* = 20.2 Hz). ^{31}P{^{1}H} δ = 42.29. ATR-IR = 2101 cm^{-1} (N_2).

Synthesis of (^{Mes}CCC)Fe(H)(PPh_3)(N_2) (2-PPh_3). A 15 mL Schlenk flask equipped with a stir bar was charged with (^{Mes}CCC)FeMes(PPh_3) (0.040 g, 0.041 mmol, 1.0 equiv.) and THF (4 mL). The flask was taken out of the glovebox and subjected to two freeze-pump-thaw cycles prior to addition of 1 atm of H_2 at 77 K. The flask was reintroduced to the glovebox and set to stir overnight to ensure full conversion. After stirring, the solution was filtered over Celite and volatiles removed under reduced pressure. The solid residue was washed with HMDSO (3 x 3 mL) and diethyl ether (2 x 1 mL), and lyophilized from benzene to give a bright orange powder in good yield (0.030 g, 0.034 mmol, 83%). Crystals suitable for X-ray diffraction were grown from a concentrated diethyl ether solution of the product with 1 drop of HMDSO. Anal. Calcd. for $C_{56}H_{49}FeN_6P$: C, 75.33; H, 5.53; N, 9.41. Found C, 75.15; H, 5.35; N, 9.38. NMR data (C_6D_6, 25 °C): ^{1}H δ = 7.78 (d, *J* = 8.0 Hz, 2H), 7.40 (d, *J* = 7.7 Hz, 2H), 7.23 (t, *J* = 7.7 Hz, 1H) 7.11 (t, *J* = 7.7 Hz, 2H), 6.98 (t, *J* = 8.1

Hz, 6H), 6.92 (t, J = 7.6 Hz, 2H), 6.84 (t, J = 7.3 Hz, 3H), 6.79 (s, 2H), 6.76 – 6.71 (m, 8H), 6.59 (d, J = 7.8 Hz, 2H), 2.08 (s, 6H), 2.03 (s, 6H), 1.63 (s, 6H), -10.92 (d, J = 22.2 Hz, 1H). $^{13}C\{^1H\}$ δ = 228.06 (d, J = 10.5 Hz), 147.34, 138.93, 138.72, 138.52, 137.63, 137.46, 136.54, 134.42, 133.43, 133.36, 132.78, 130.18, 128.96, 127.51, 127.46, 121.95, 121.62, 120.47, 109.61, 108.64, 106.13, 21.09, 18.62, 17.86. $^{31}P\{^1H\}$ δ = 49.13. ATR-IR = 2096 cm^{-1} ($Fe-N_2$).

NMR spectra of Metal Complexes

NMR spectra of (**^{Mes}CCC)Fe(H)(Py)(N_2)**

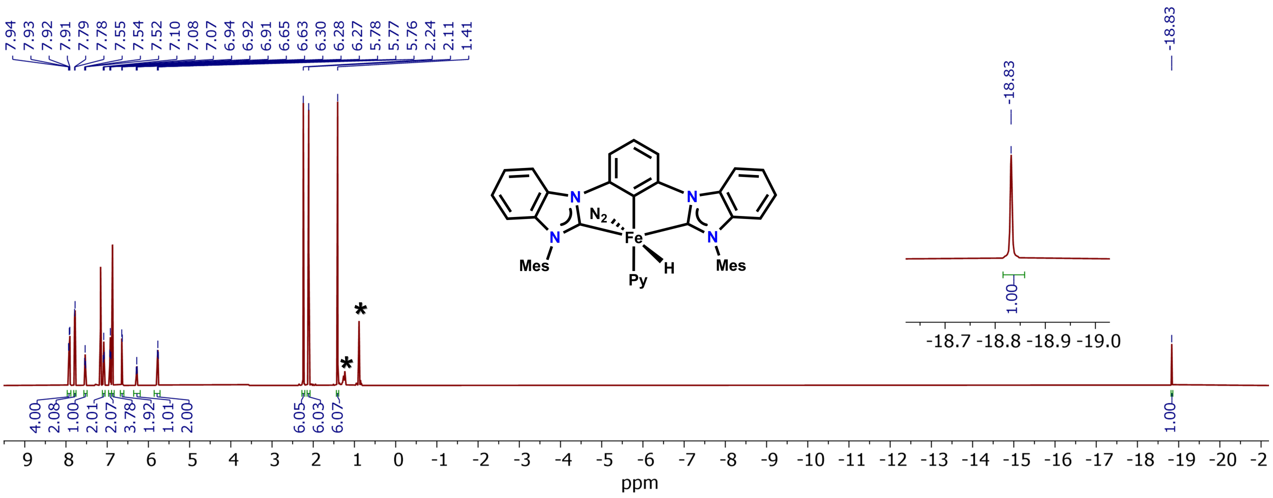

Figure S1. ^1H NMR spectrum of **(^{Mes}CCC)Fe(H)(Py)(N_2)** in C_6D_6. (*) Denotes hexane.

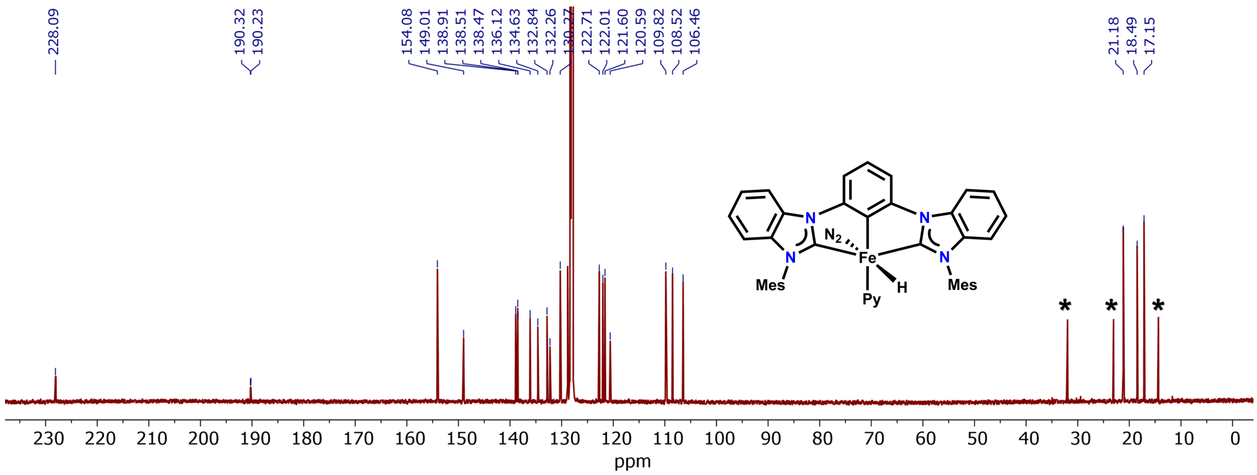

Figure S2. $^{13}C\{^1H\}$ NMR spectrum of **(^{Mes}CCC)Fe(H)(Py)(N_2)** in C_6D_6. (*) Denotes hexane.

NMR spectra of (^{Mes}CCC)Fe(H)(PMe$_3$)(N$_2$)

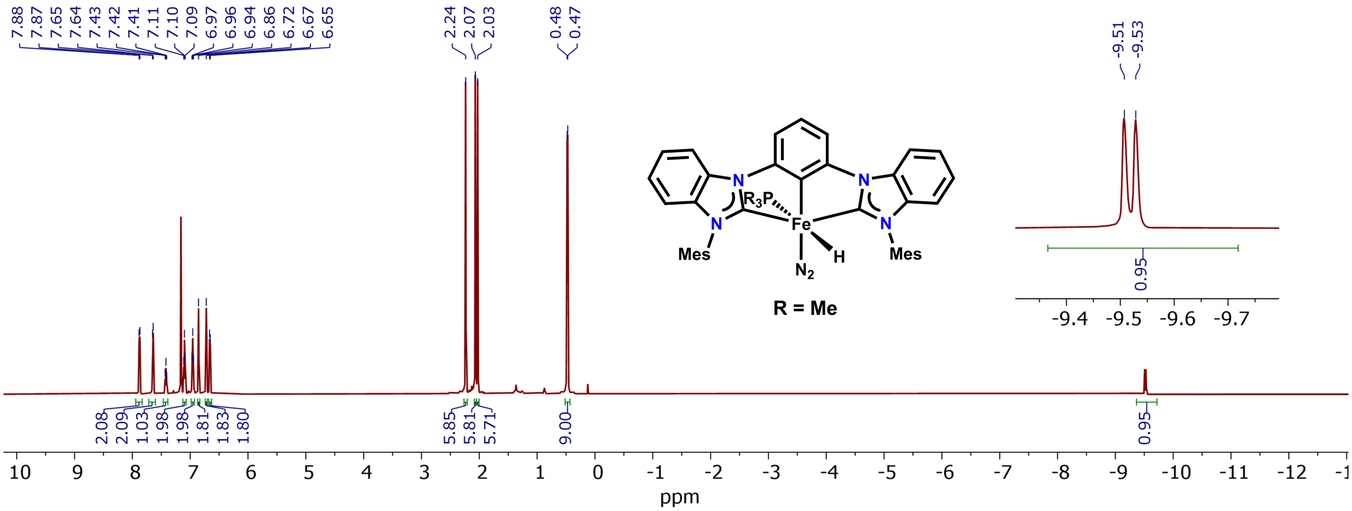

Figure S3. ^{1}H NMR spectrum of **(^{Mes}CCC)Fe(H)(PMe$_3$)(N$_2$)** in C_6D_6.

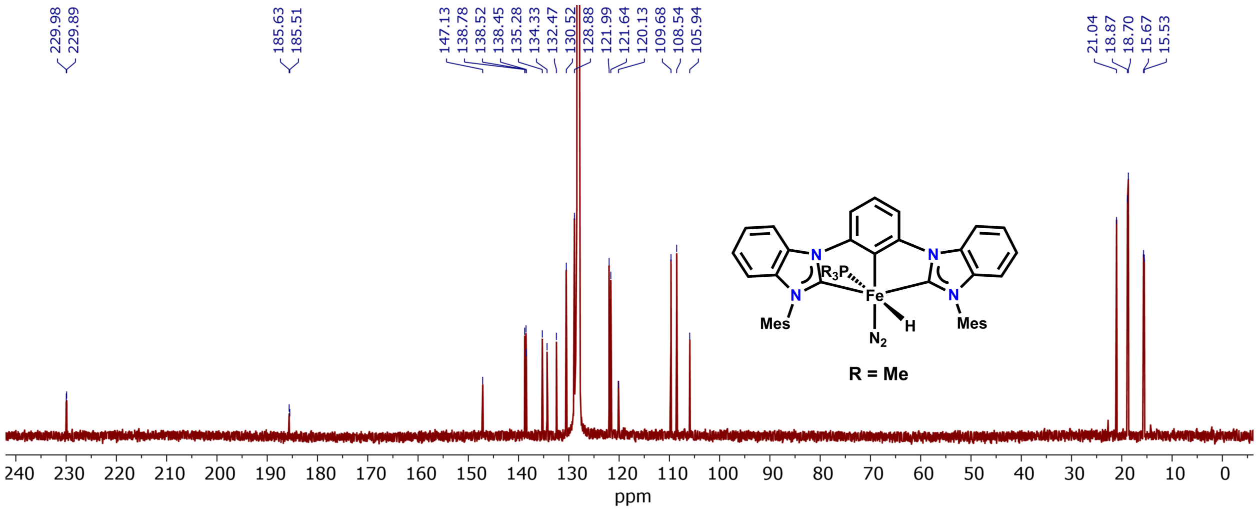

Figure S4. ^{13}C{^{1}H} NMR spectrum of **(^{Mes}CCC)Fe(H)(PMe$_3$)(N$_2$)** in C_6D_6.

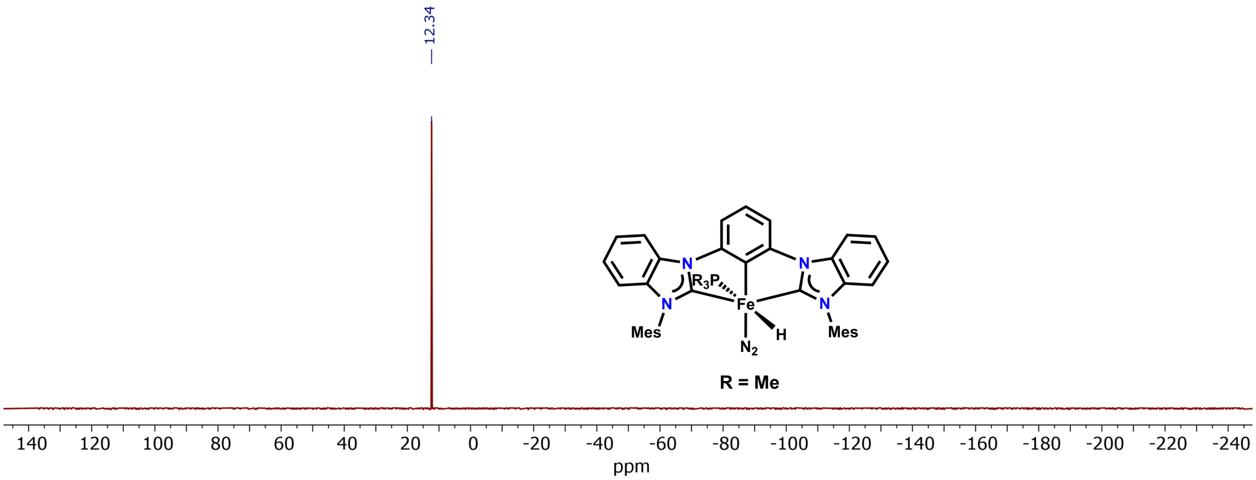

Figure S5. $^{31}P\{^{1}H\}$ NMR spectrum of **(^{Mes}CCC)Fe(H)(PMe$_3$)(N$_2$)** in C_6D_6.

NMR spectra of (^{Mes}CCC)Fe(H)(PPh$_3$)(N$_2$)

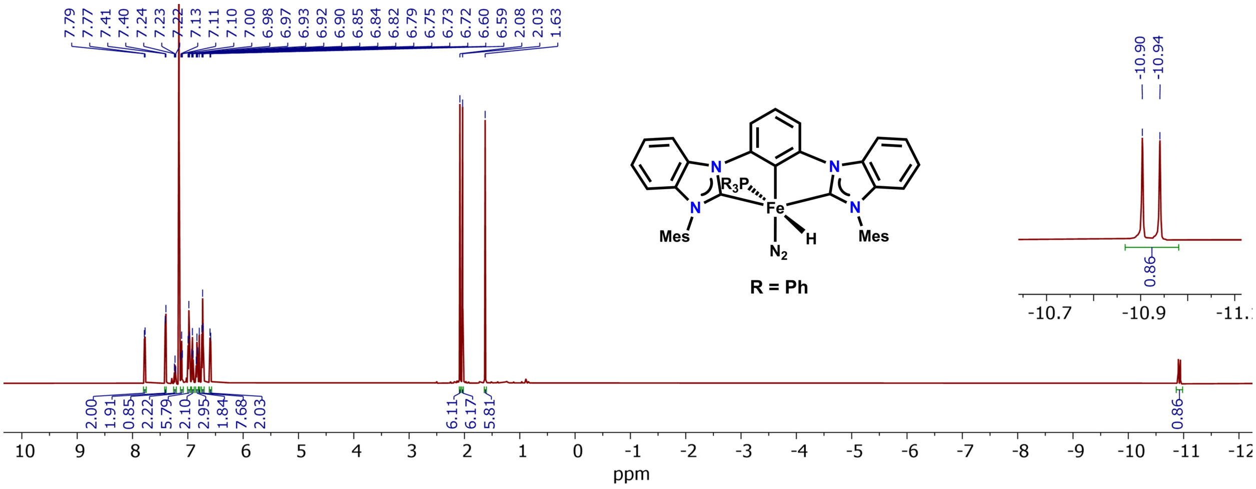

Figure S6. ^{1}H NMR spectrum of **(^{Mes}CCC)Fe(H)(PPh$_3$)(N$_2$)** in C_6D_6.

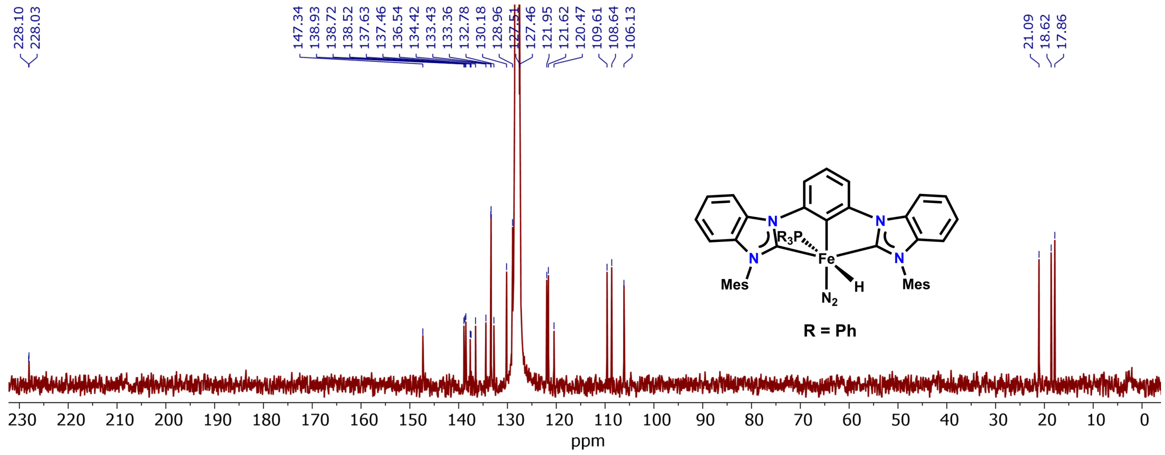

Figure S7. $^{13}C\{^1H\}$ NMR spectrum of **(^{Mes}CCC)Fe(H)(PPh$_3$)(N$_2$)** in C_6D_6.

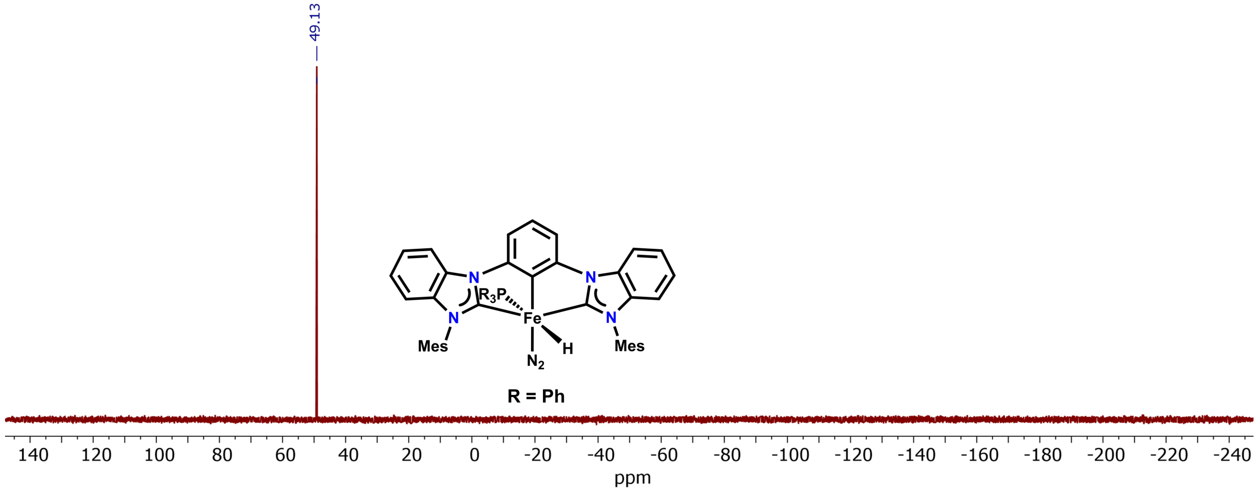

Figure S8. $^{31}P\{^1H\}$ NMR spectrum of **(^{Mes}CCC)Fe(H)(PPh$_3$)(N$_2$)** in C_6D_6.

Reaction of 1-Py with D_2: synthesis of (^{Mes}CCC)Fe(D)(Py)(N_2)

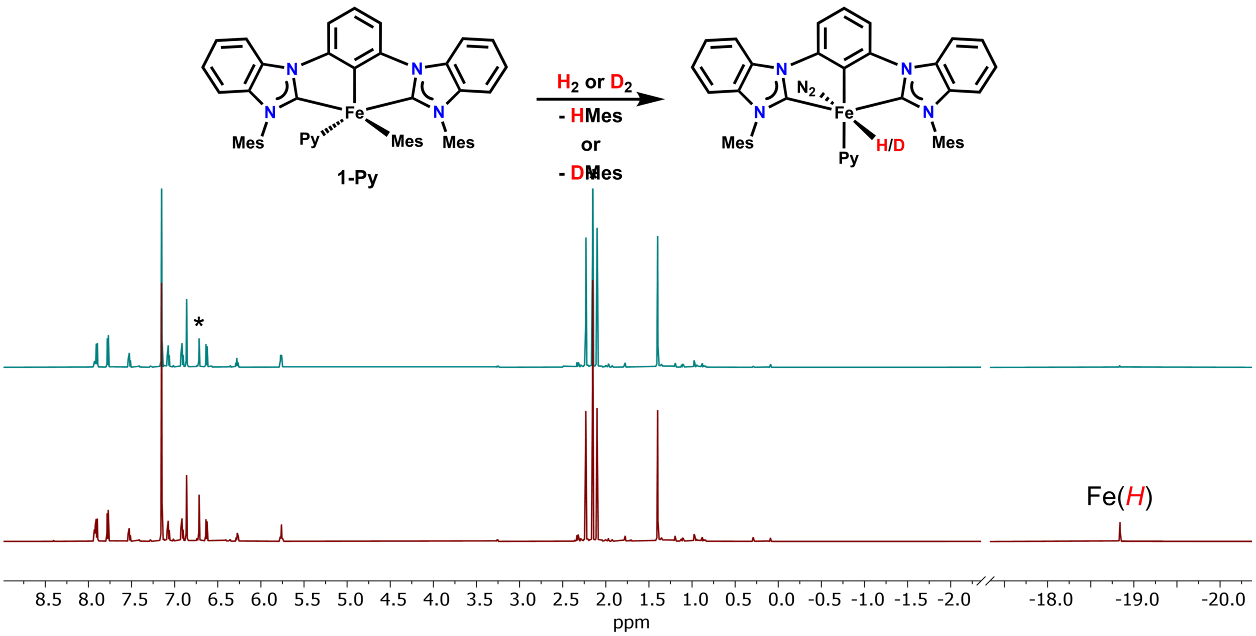

Figure S9. ^{1}H NMR spectrum of the reaction of **1-Py** with D_2 to yield (^{Mes}CCC)Fe(D)(Py)(N_2) (top); and ^{1}H NMR spectrum of the reaction of **1-Py** with H_2 to yield (^{Mes}CCC)Fe(H)(Py)(N_2) (**2-Py**) (bottom), all in C_6D_6. (*) Denotes mesitylene.

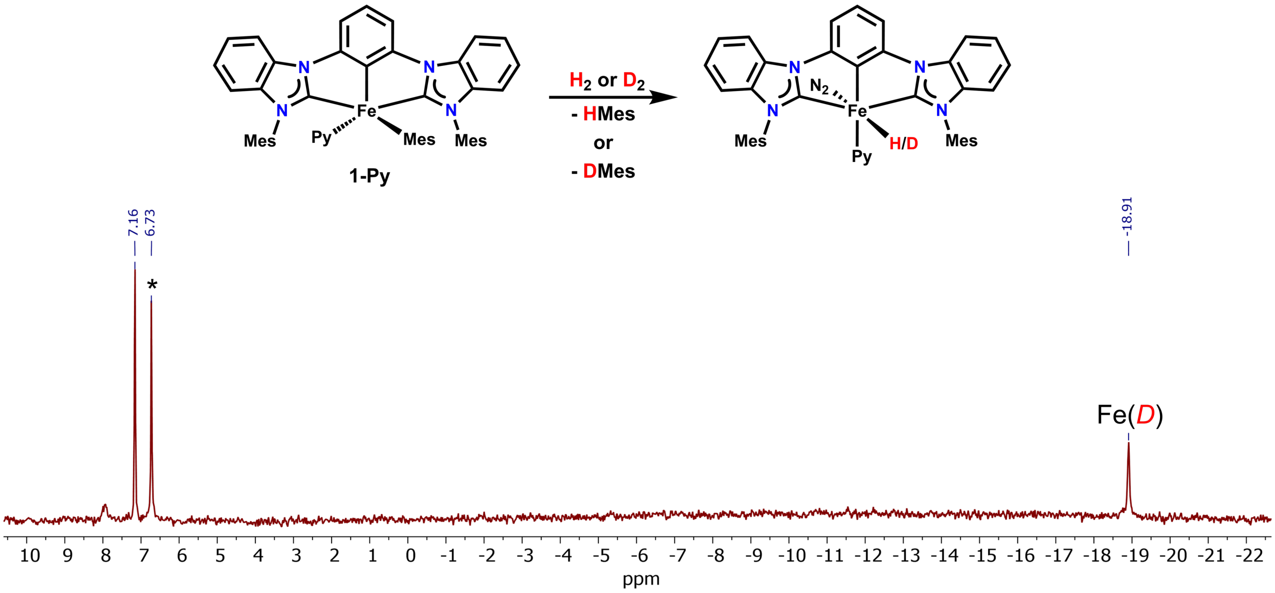

Figure S10. ^{2}H NMR spectrum of the reaction of **1-Py** with D_2 to yield (^{Mes}CCC)Fe(D)(Py)(N_2) in C_6H_6. (*) Denotes mesitylene-d_1.

Stability of 2-Py in solution

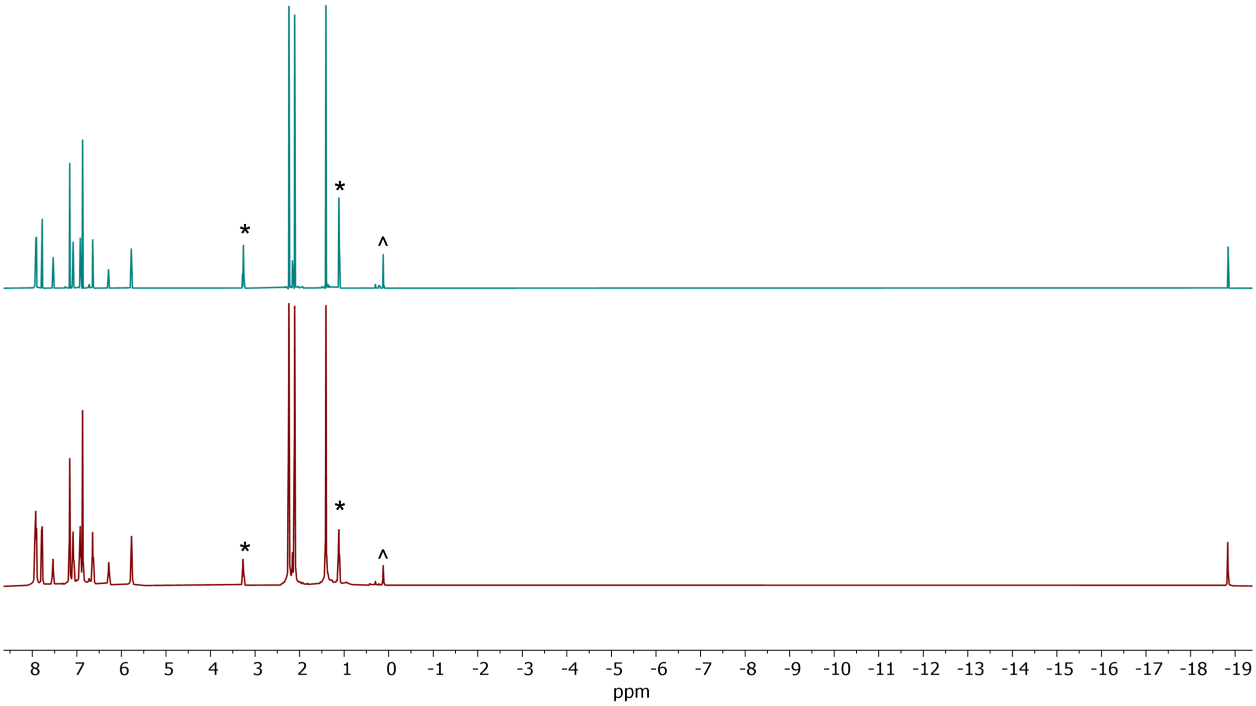

Figure S11. ^{1}H NMR spectra of a 10mM solution of **2-Py** (top); and the same sample after 30 days at room temperature (bottom), all in C_6D_6. (*) Denotes diethyl ether, (^) denotes HMDSO.

Olefin hydrogenation with 2-Py

General hydrogenation procedure. To a 4 mL scintillation vial were added **2-Py** (2.1 mg, 0.0029 mmol, 2 mol%), mesitylene (0.144 mmol, 1 equiv.), and substrate (0.144 mmol, 1 equiv.) and 0.5 mL of C_6D_6. The mixture was transferred to a J. Young NMR tube and taken out of the glovebox. The sample was subjected to two freeze-pump-thaw cycles. After a third pump stage, 1 atm of H_2 was added at 77 K resulting in 4 atm of H_2 at room temperature. After thawing, the sample was secured with Parafilm to a 14/20 solvent trap connected to a rotary evaporator and subjected to repeated inversion on the lowest rotation setting. Conversion to the product was monitored by ^{1}H NMR spectroscopy. Control experiments with other (^{Mes}CCC)Fe complexes were carried out under similar conditions.

NMR Spectra of Hydrogenation Reactions

Styrene:

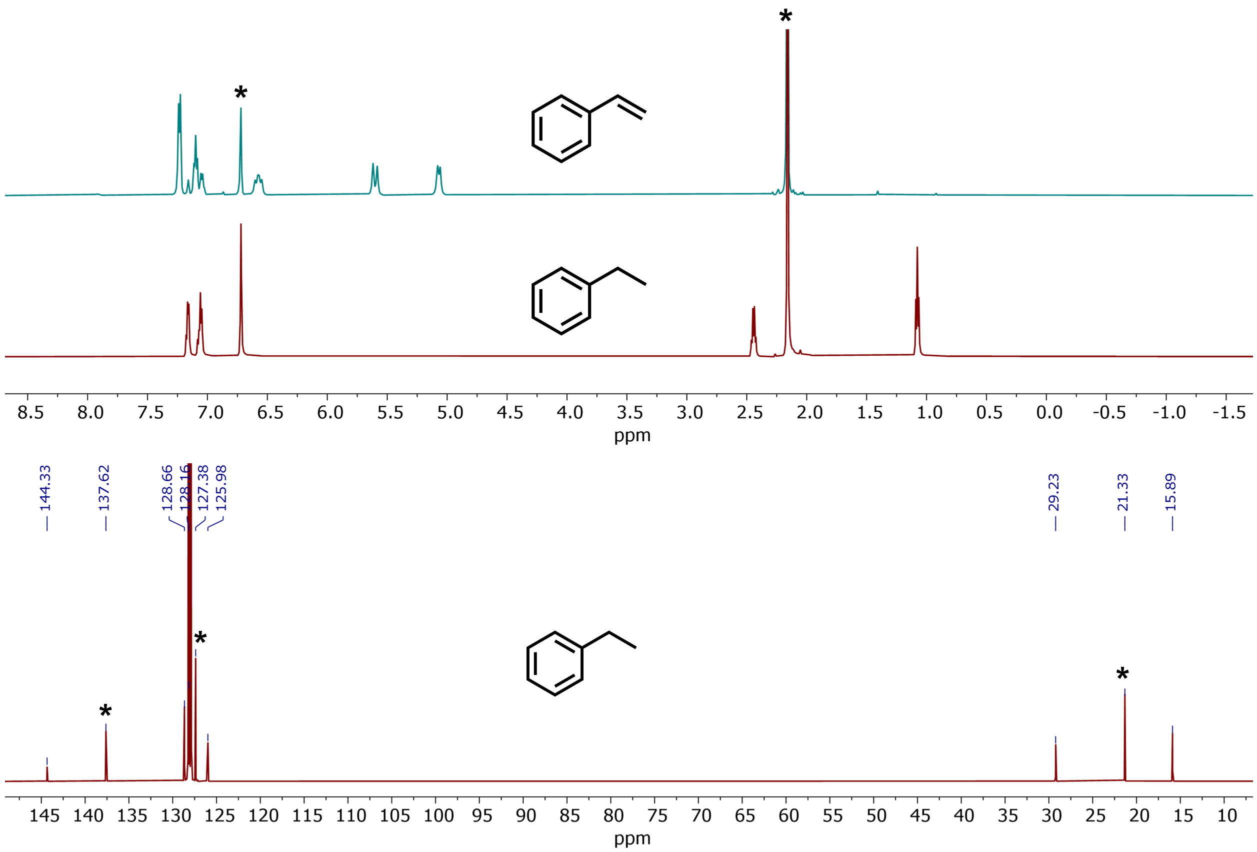

Figure S12. ^{1}H NMR spectra of **2-Py**, styrene, and mesitylene prior to H_2 addition and after 30 min under 4 atm of H_2 (top); and $^{13}C\{^1H\}$ NMR spectrum after hydrogenation (bottom), all in C_6D_6. (*) Denotes mesitylene.

4-Methoxystyrene:

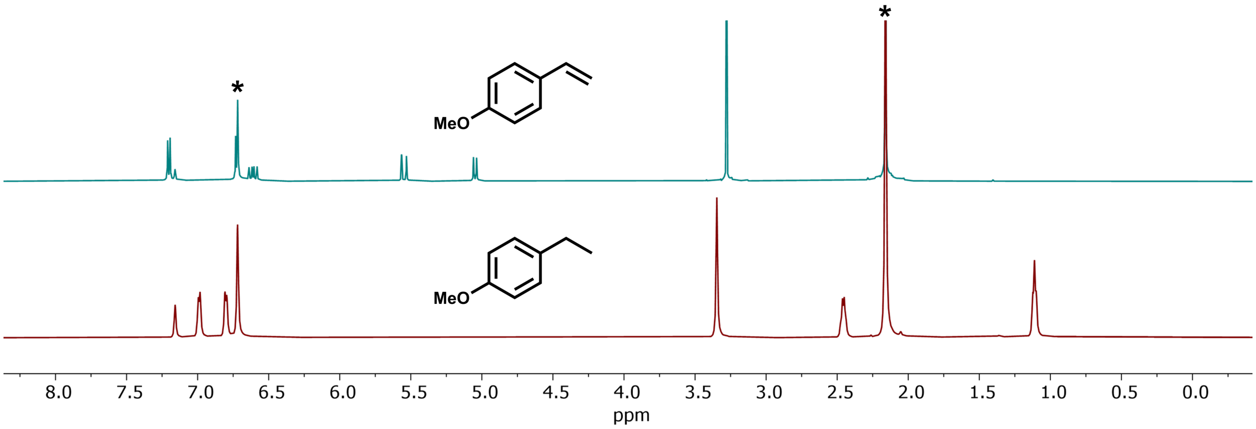

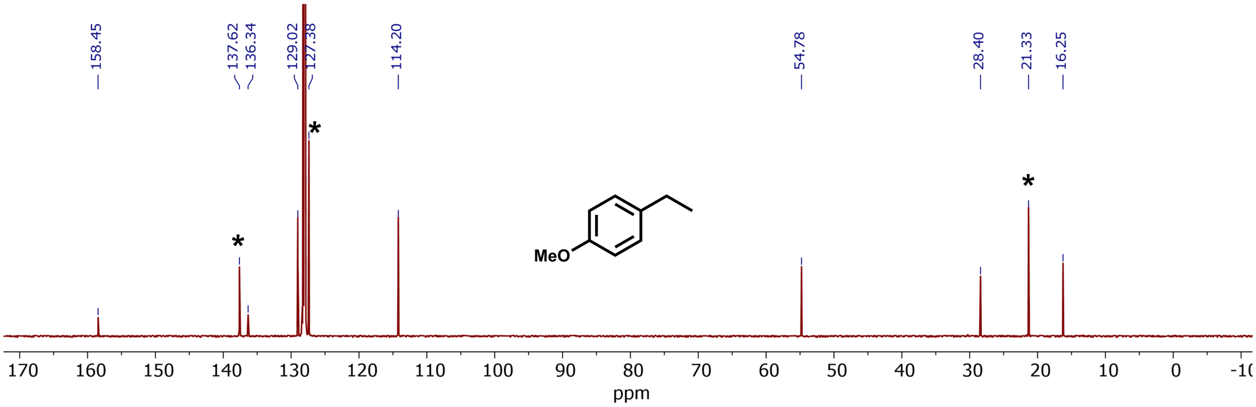

Figure S13. ^{1}H NMR spectra of **2-Py**, 4-methoxystyrene, and mesitylene prior to H_2 addition and after 30 min under 4 atm of H_2 (top); $^{13}C\{^{1}H\}$ NMR spectrum after hydrogenation (middle); and ^{19}F NMR spectrum after hydrogenation, all in C_6D_6. (*) Denotes mesitylene.

4-Fluoroystyrene:

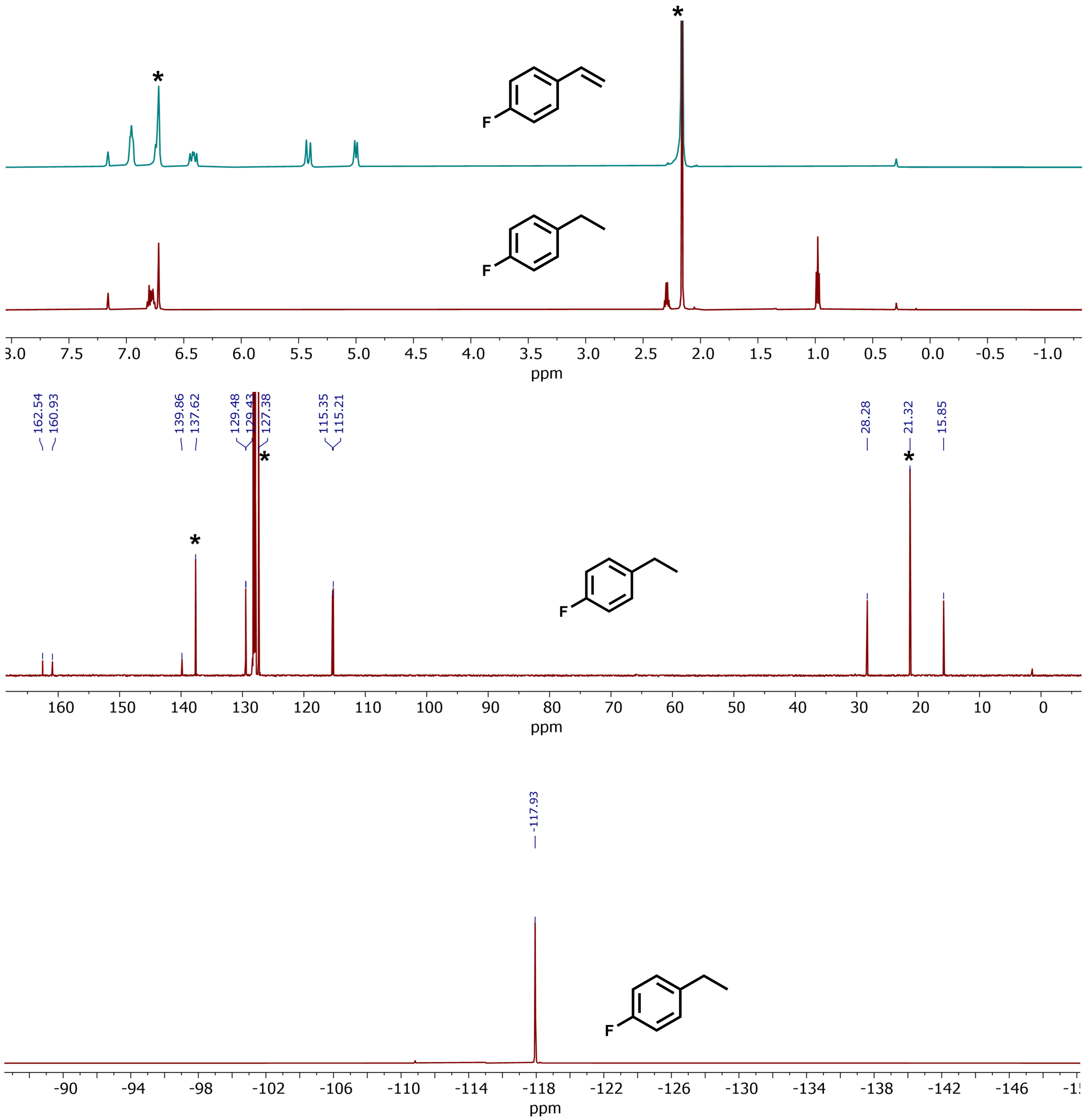

Figure S14. ^{1}H NMR spectra of **2-Py**, 4-fluorostyrene, and mesitylene prior to H_2 addition and after 2 h under 4 atm of H_2 (top); $^{13}C\{^{1}H\}$ NMR spectrum after hydrogenation (middle); and ^{19}F NMR spectrum after hydrogenation, all in C_6D_6. (*) Denotes mesitylene.

1-Octene

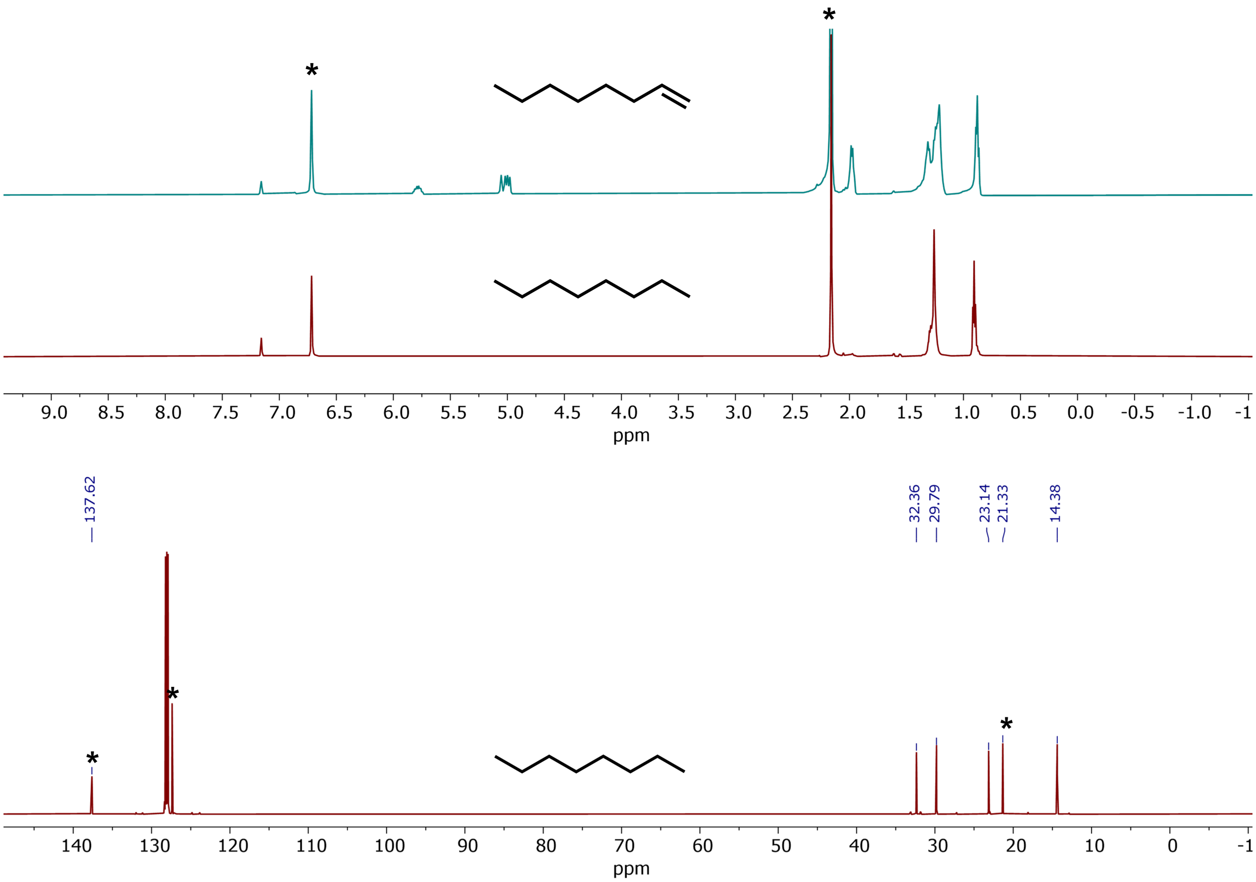

Figure S15. ^{1}H NMR spectra of **2-Py**, 1-octene, and mesitylene prior to H_2 addition and after 1 h under 4 atm of H_2 (top); $^{13}C\{^1H\}$ NMR spectrum after hydrogenation (bottom), all in C_6D_6. (*) Denotes mesitylene.

4-vinylcyclohexene:

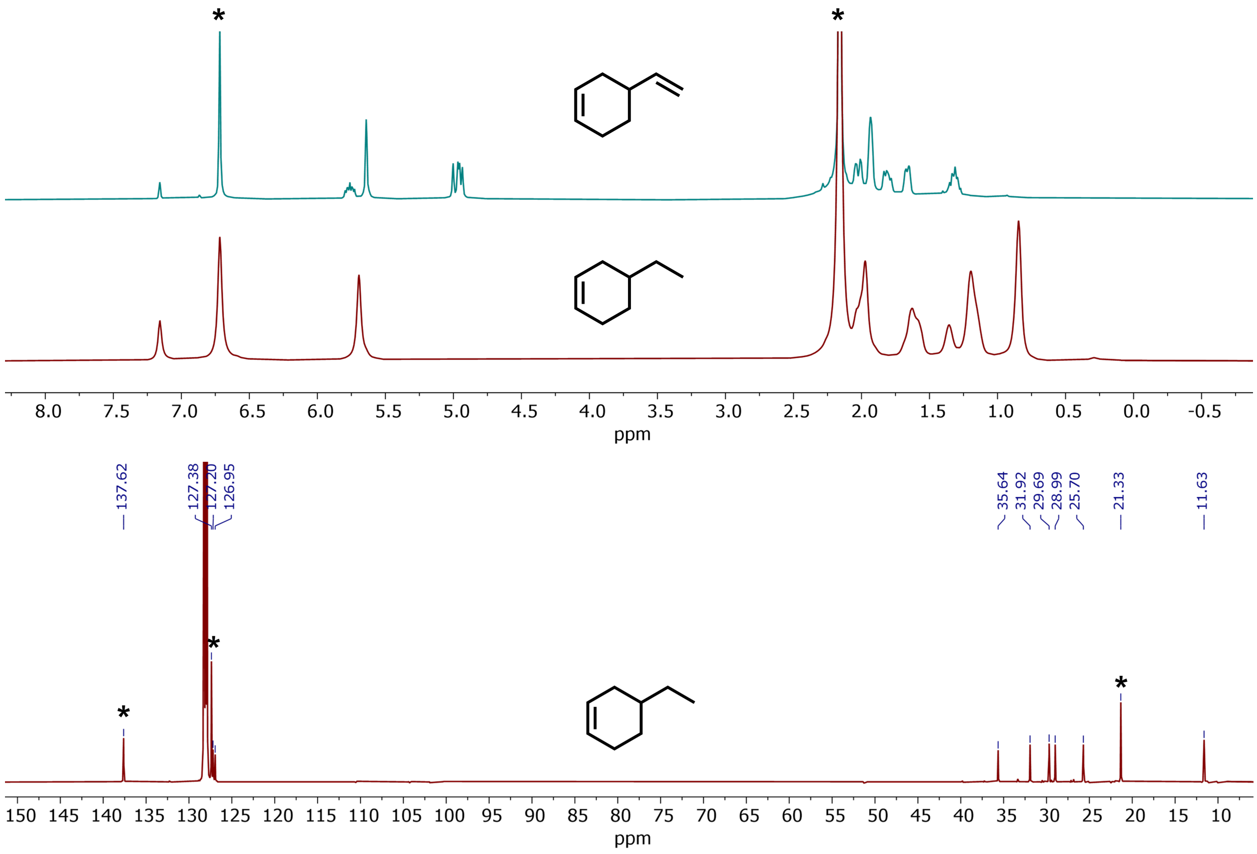

Figure S16. ^{1}H NMR spectra of **2-Py**, 4-vinylcyclohexene, and mesitylene prior to H_2 addition and after 1 h under 4 atm of H_2 (top); $^{13}C\{^{1}H\}$ NMR spectrum after hydrogenation (bottom), all in C_6D_6. (*) Denotes mesitylene.

Vinyltrimethylsilane

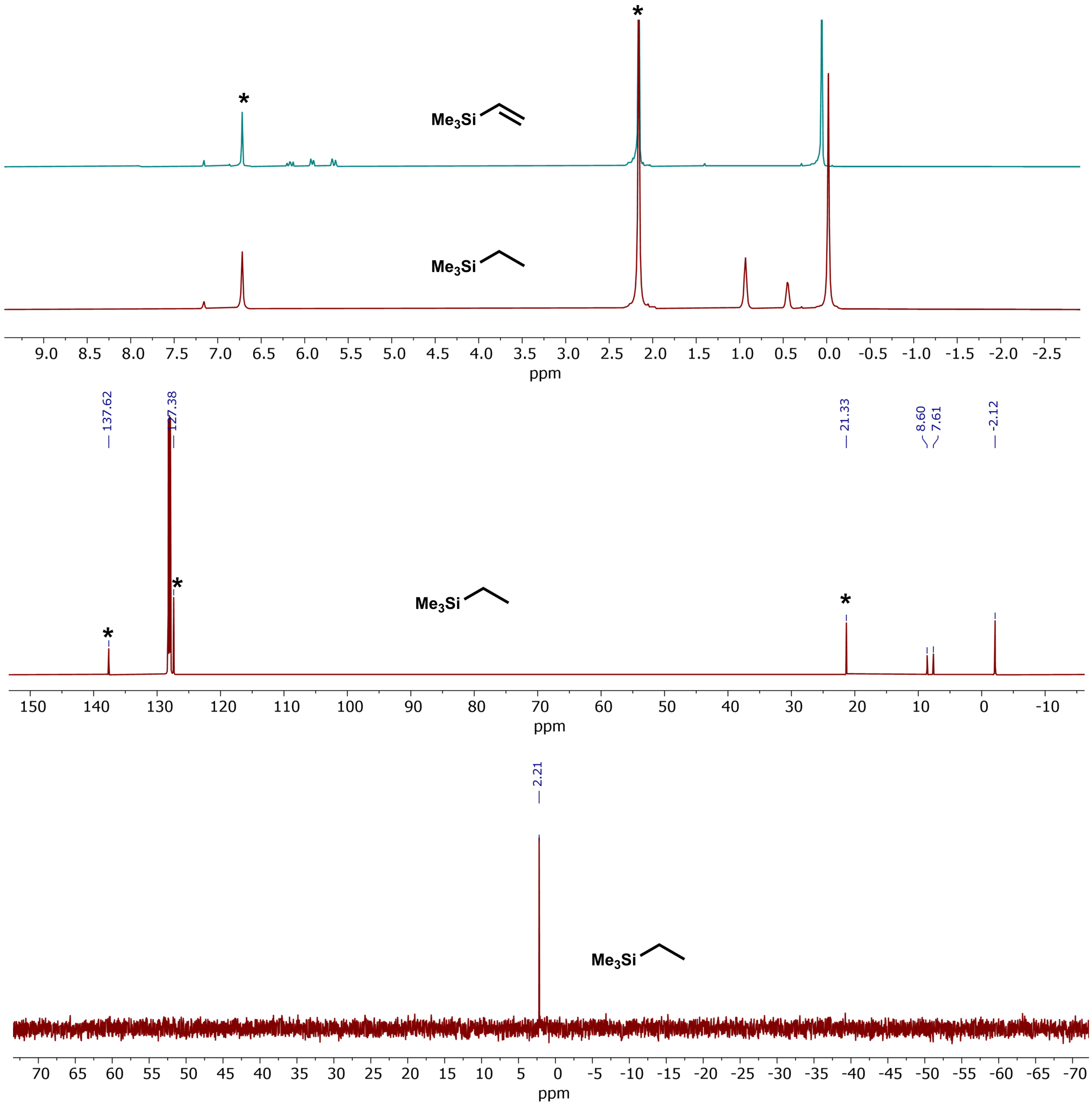

Figure S17. ^{1}H NMR spectra of **2-Py**, vinyltrimethylsilane, and mesitylene prior to H_2 addition and after 2 h under 4 atm of H_2 (top); $^{13}C\{^{1}H\}$ NMR spectrum after hydrogenation (middle); and $^{29}Si\{^{1}H\}$ NMR spectrum after hydrogenation, all in C_6D_6. (*) Denotes mesitylene.

Methyl-3,3-dimethylpentenoate:

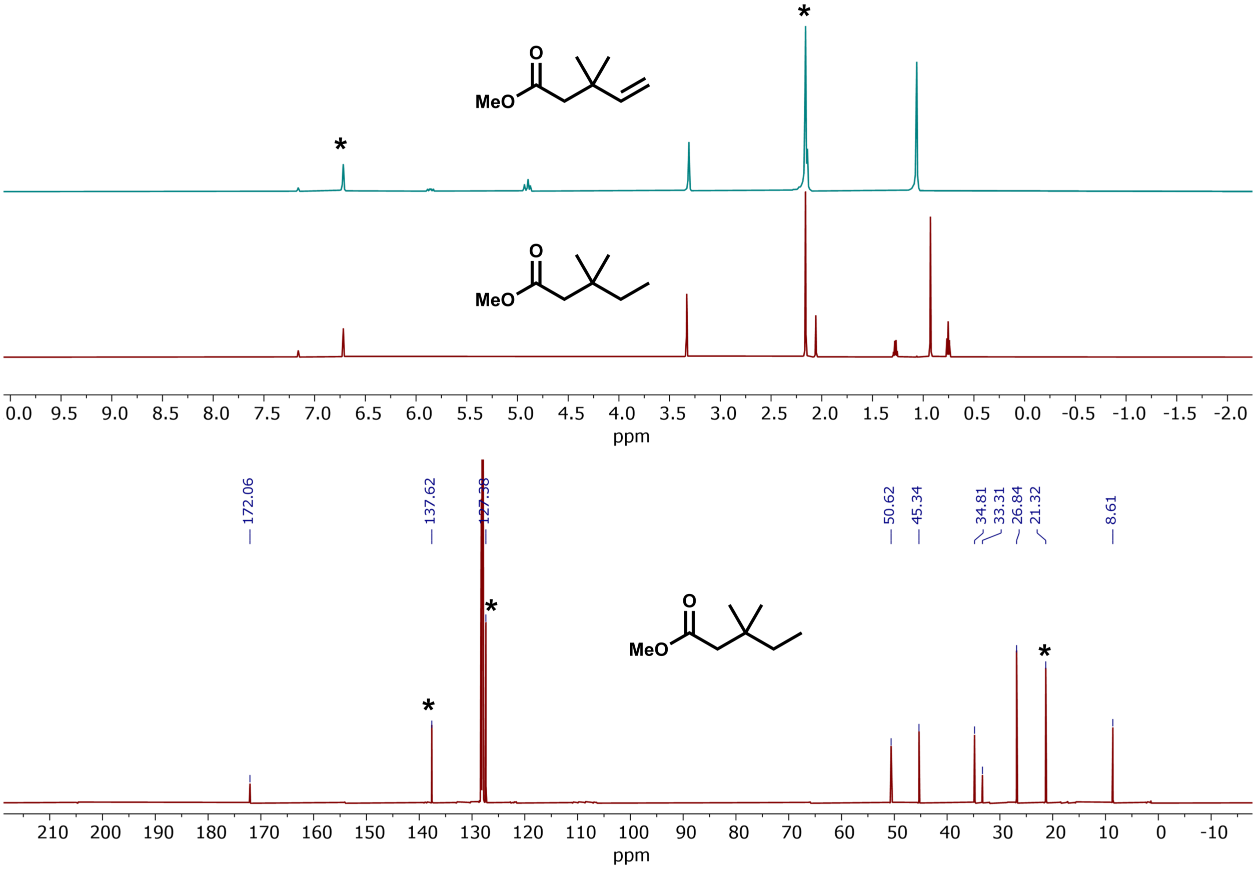

Figure S18. ^{1}H NMR spectra of **2-Py**, methyl-3,3-dimethylpentenoate, and mesitylene prior to H_2 addition and after 20 h under 4 atm of H_2 (top); $^{13}C\{^1H\}$ NMR spectrum after hydrogenation (bottom), all in C_6D_6. (*) Denotes mesitylene.

Deuteration experiments

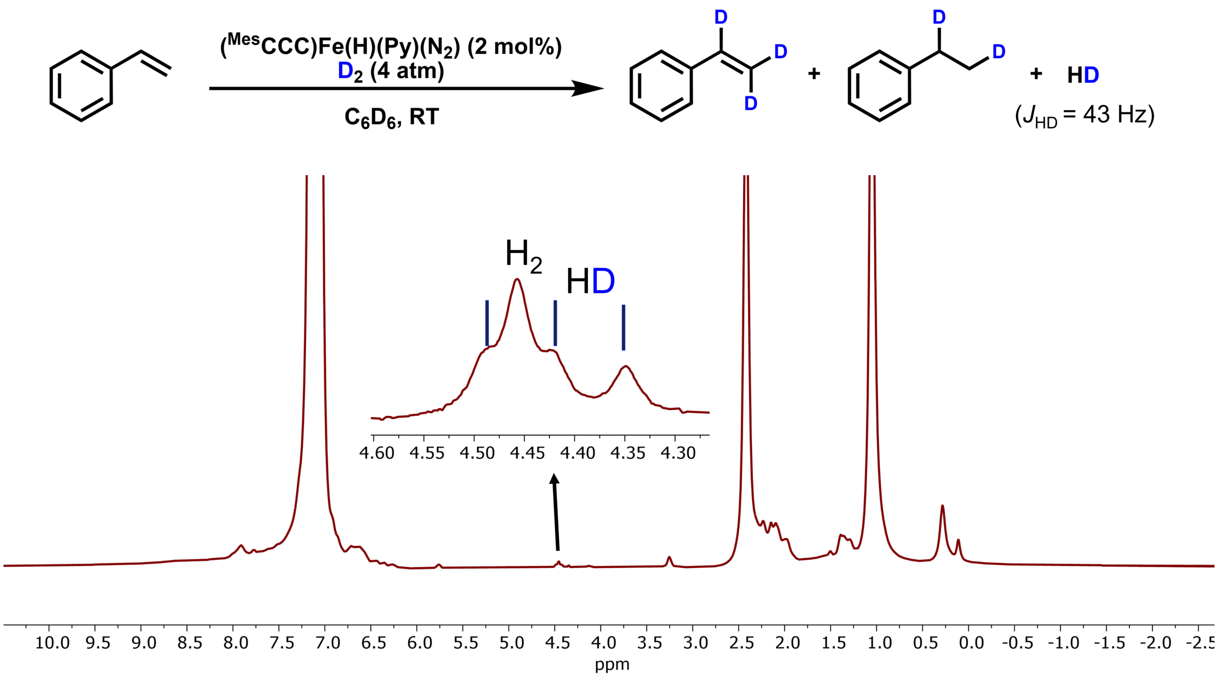

Figure S19 ^{1}H NMR spectrum of the reaction of styrene with D_2 (4 atm) in the presence of **2-Py** in C_6D_6 after 2 h. Inset shows the formation of H_2 and HD gas under catalytic conditions.

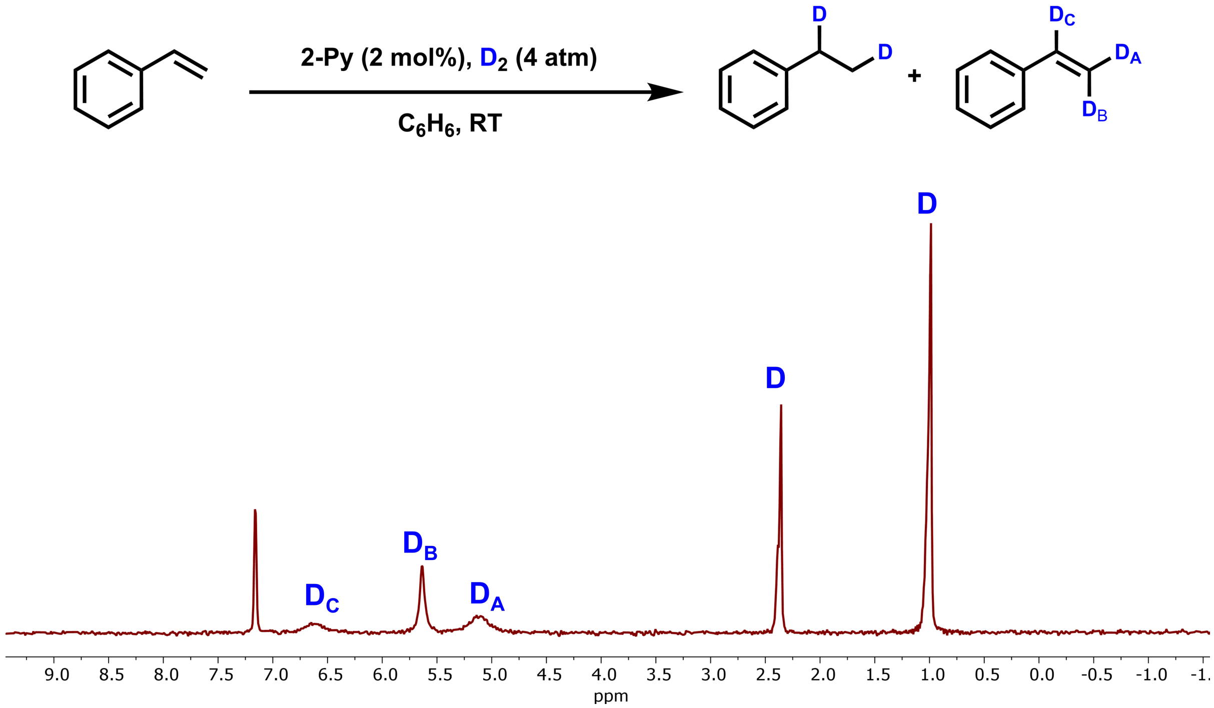

Figure S20 ^{2}H NMR spectrum of **2-Py** and styrene in C_6H_6 5 minutes after D_2 addition.

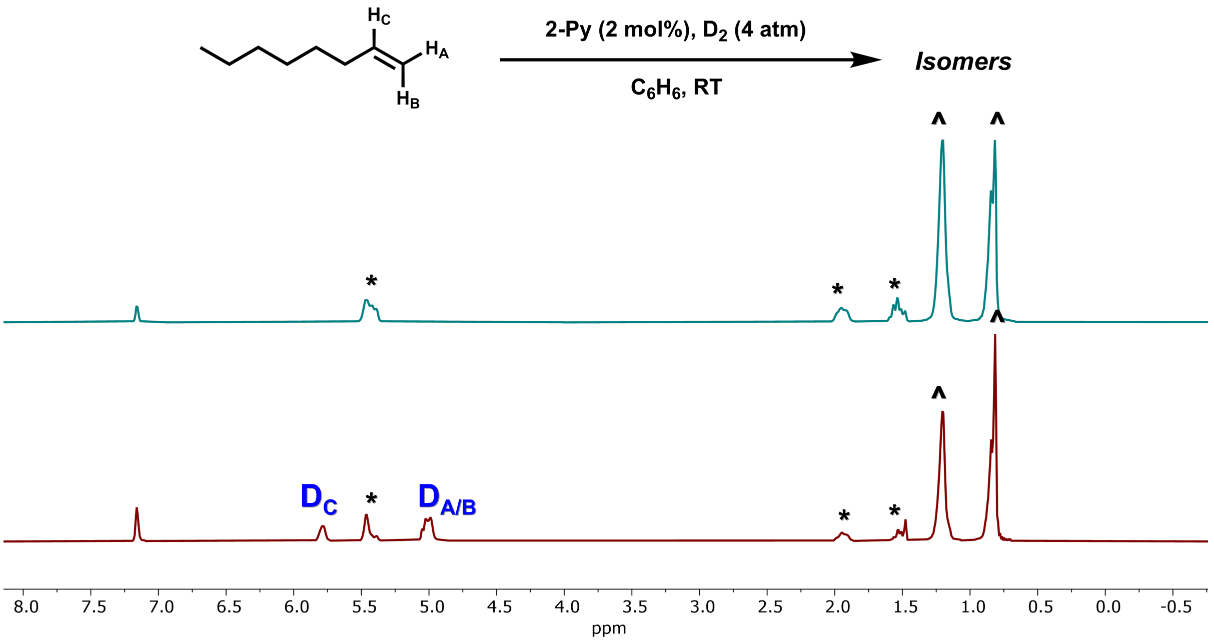

Figure S21 ^{2}H NMR spectra of **2-Py** and 1-octene in C_6H_6 5 minutes after D_2 addition (bottom); and after 4 h (top). *Denotes deuterated internal alkene, (^) denotes octane-d_2.

Isomerization of 1-octene

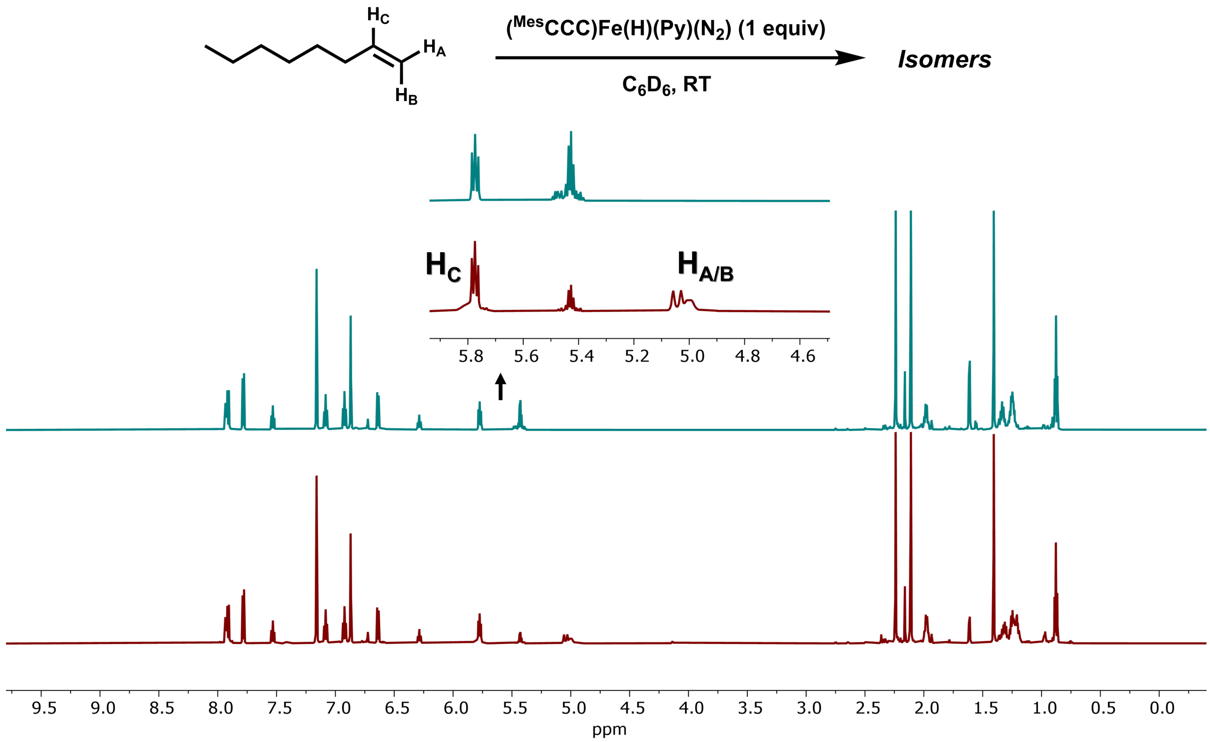

Figure S22 ^{1}H NMR spectra of the stoichiometric reaction of 1-octene and **2-Py** in C_6D_6 after 30 min (bottom) and 2 h (top). Inset shows the relevant olefinic resonances of 1-octene at 5.01 ppm and the isomerization products between 5.36-5.51 ppm. The 1-octene resonance H_C overlaps with a triplet in **2-Py** that is observed after the reaction.

Para-Hydrogen Studies

Sample Preparation. To a 4 mL scintillation vial were added **2-Py** (3.4 mg, 0.00474 mmol) and 0.70 mL of THF-d_8. Substrate (0.0474 mmol) was added to the vial and the resulting mixture transferred to a J. Young NMR tube. The sample was subjected to two freeze-pump-thaw cycles. After a third pump step, 1 atm of *para*-hydrogen was added while the sample was frozen in liquid nitrogen. The sample was kept in liquid nitrogen and thawed in an isopropanol bath prior to careful insertion into the NMR spectrometer regulated to 25 °C. After shimming, the samples were ejected from the NMR spectrometer and shaken immediately for 5 seconds before being reintroduced into the instrument for analysis. The samples were shaken right outside the spectrometer with a field measured at about 50 G (5.0×10^{-3} T) with a cellphone gaussmeter (Gauss Meter).

NMR Spectrometer. 1H NMR spectra were collected on a Varian UNITY INOVA 600 NB High Resolution NMR Console (Ui600) with a 5mm Varian AutoTuneX ^{1}H/X PFG Z probe, X=^{31}P, ^{15}N. All ^{1}H NMR spectra were collected in THF-d_8 and the residual solvent resonance was referenced to 3.58 ppm. Spectra were recorded using 90° and 45° pulse angles.

Generation of parahydrogen. A *para*-hydrogen converter was used to generate the p-H_2 enriched hydrogen gas. This consisted of copper tubing filled with a hydrous ferric oxide catalyst that was cooled to ~20 K using a closed-cycle ^{4}He cryostat, which was able to consistently convert naturally occurring hydrogen gas to >90% p-H_2. A detailed description of the converter can be found in Tom et al.[8]

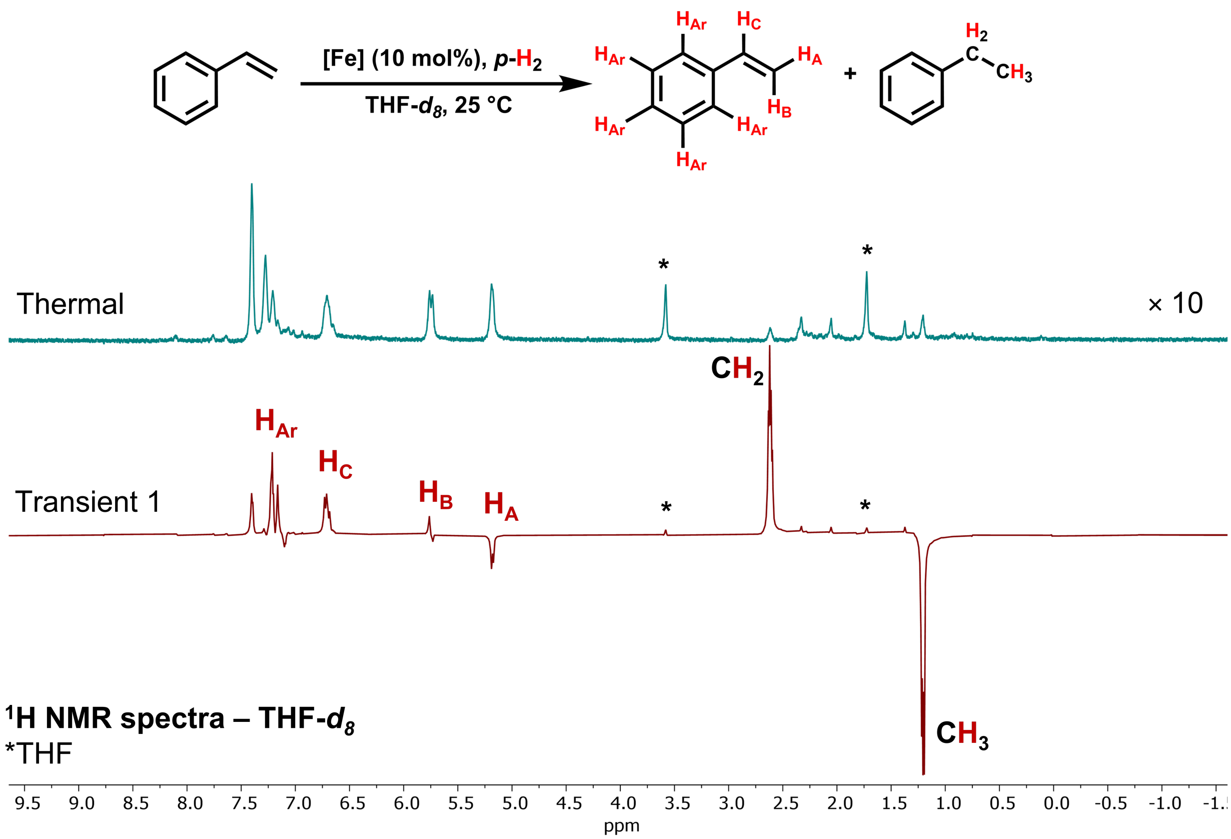

Figure S23 Normalized single transient ^{1}H NMR spectra of the reaction of styrene with *p*-H_2 (4 atm) in the presence of **2-Py**. Bottom spectrum was collected immediately following addition of *p*-H_2, while the top spectrum was taken after full relaxation of the nuclear spins back to thermal equilibrium amplified by a factor of 10.

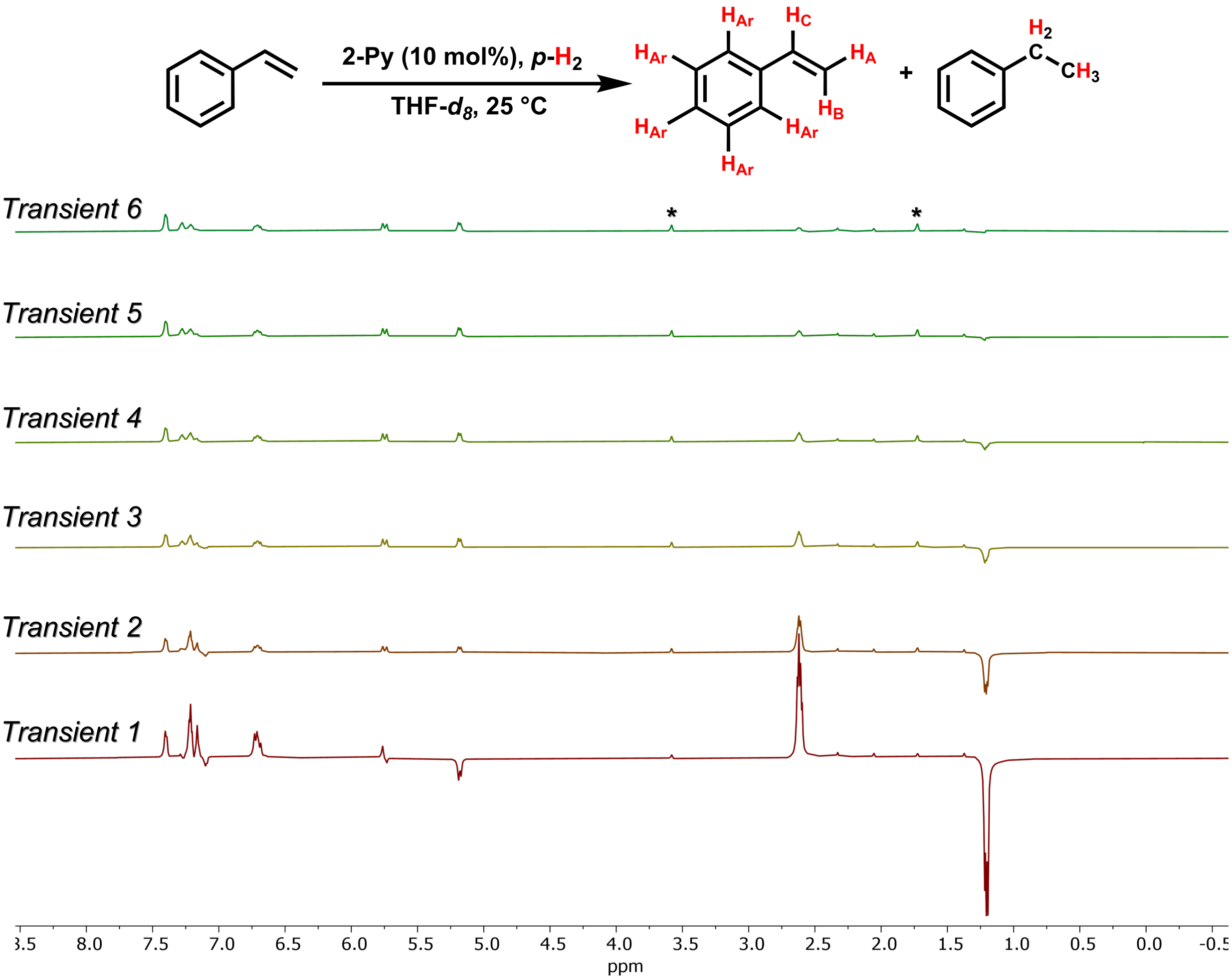

Figure S24 One-transient array of ^{1}H NMR spectra showing the decay of polarization of styrene in the reaction with *p*-H_2 and **2-Py** at the 50G fringe line of the magnet and with a 45° pulse**.** Bottom spectrum was collected immediately after shaking the sample and inserting into the instrument, followed by an acquisition time of 4.096 s before collection of each subsequent transient. Sample was only shaken once. (*)Denotes THF

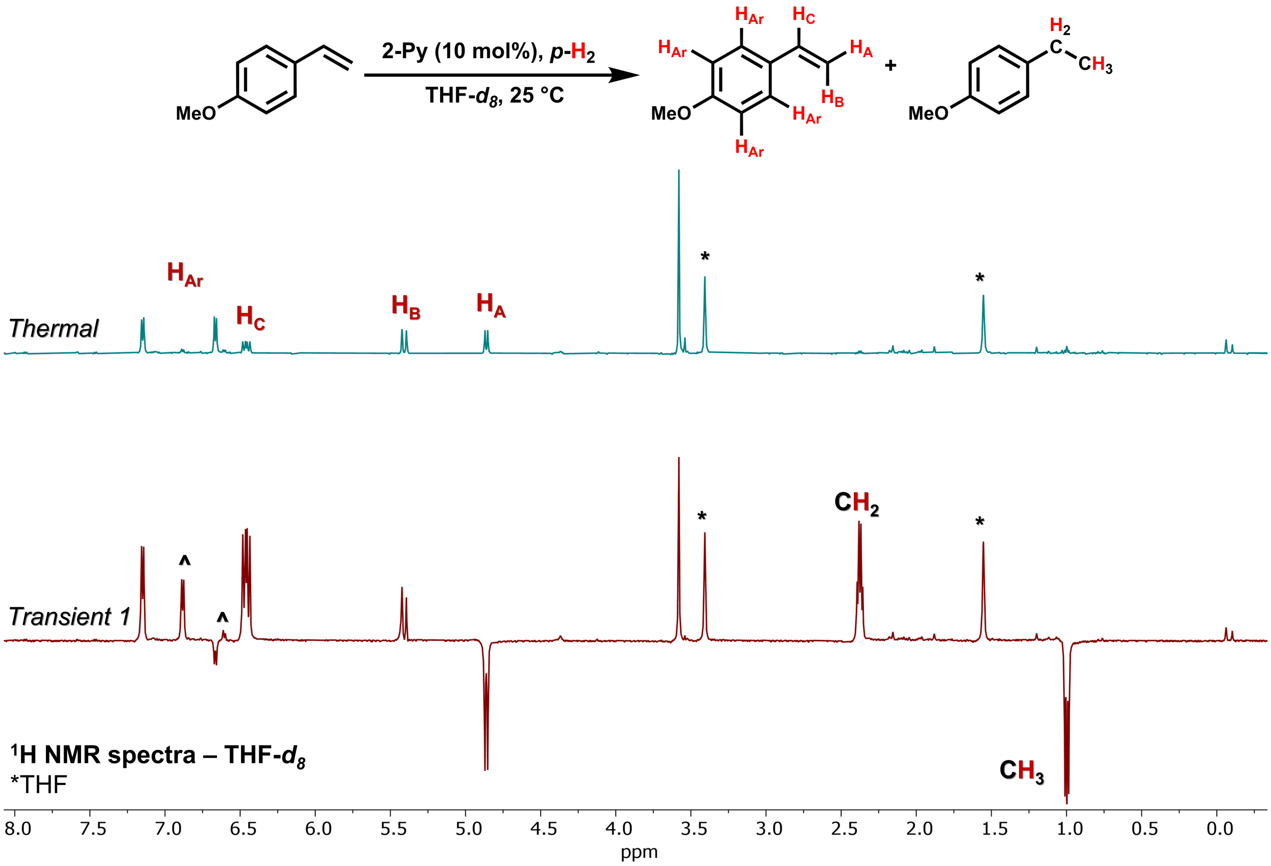

Figure S25 Normalized single transient ^{1}H NMR spectra of the reaction of 4-methoxystyrene with *p*-H_2 (4 atm) in the presence of **2-Py**. Bottom spectrum was collected immediately following addition of *p*-H_2, while the top spectrum was taken after full relaxation of the nuclear spins back to thermal equilibrium. (^) Denotes aryl resonances of the product.

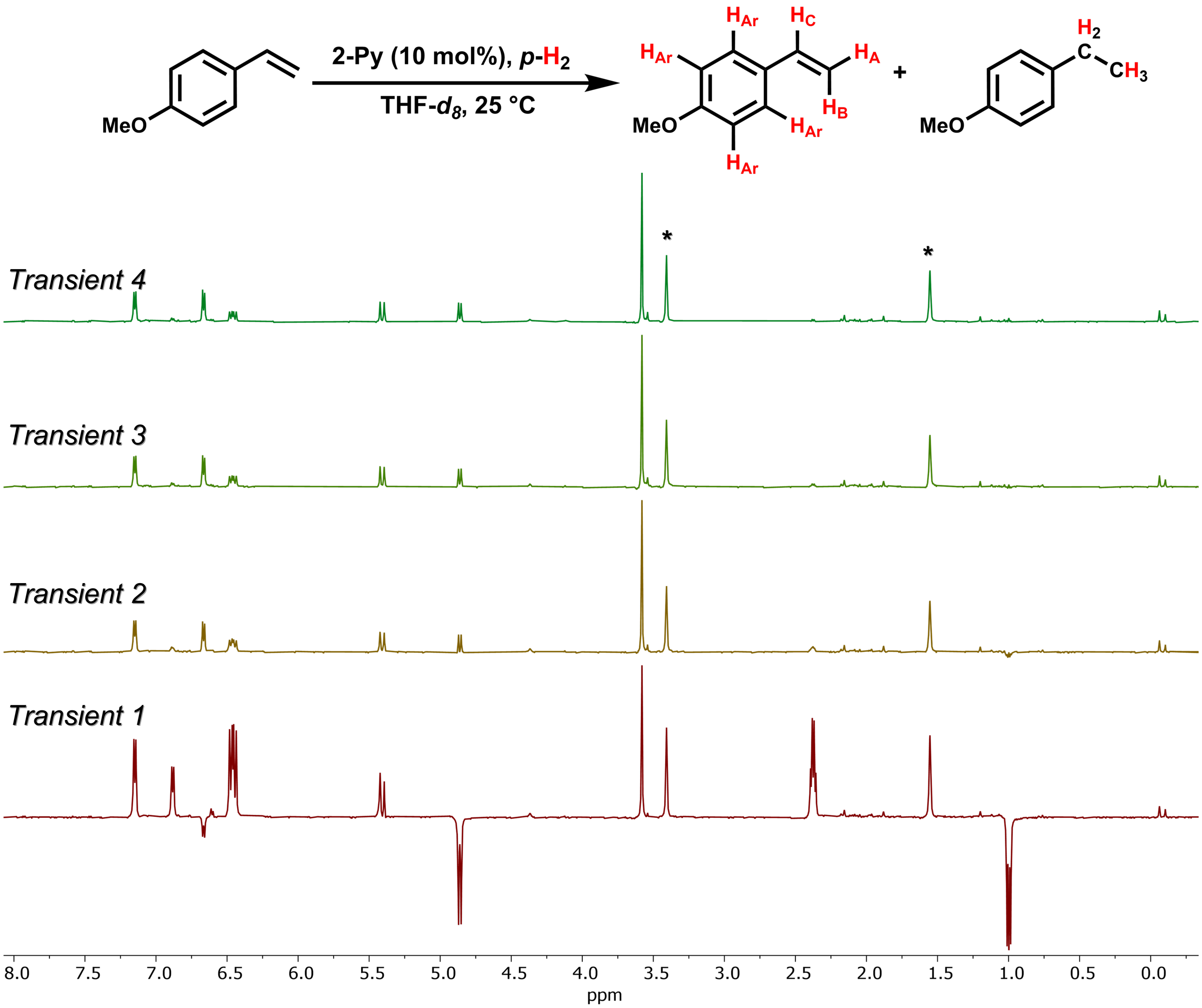

Figure S26 One-transient array of ^{1}H NMR spectra showing the decay of polarization of 4-methoxystyrene in the reaction with p-H_2 and **2-Py** at the 50G fringe line of the magnet and with a 45° pulse**.** Bottom spectrum was collected immediately after shaking the sample and inserting into the instrument, followed by an acquisition time of 4.096 s before collection of each subsequent transient. Sample was only shaken once. (*)Denotes THF

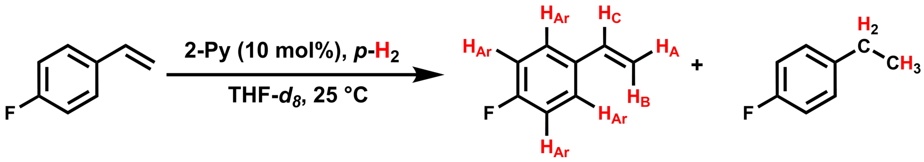

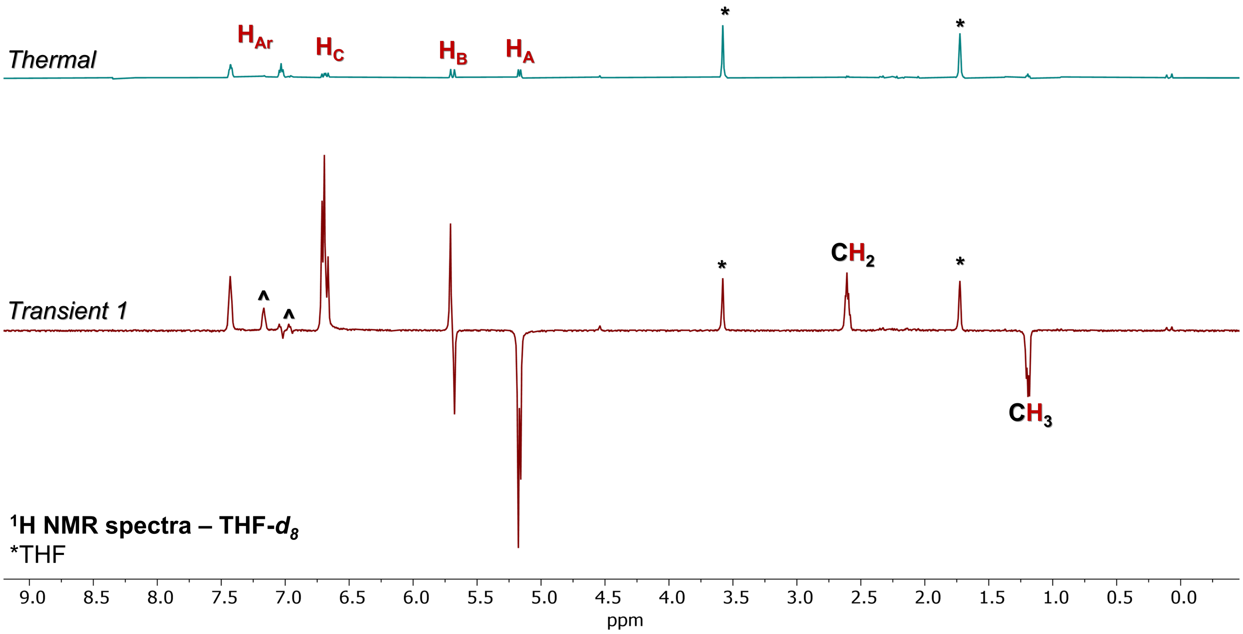

Figure S27 Normalized single transient ^{1}H NMR spectra of the reaction of 4-fluorostyrene with *p*-H_2 (4 atm) in the presence of **2-Py**. Bottom spectrum was collected immediately following addition of *p*-H_2, while the top spectrum was taken after full relaxation of the nuclear spins back to thermal equilibrium. (^) Denotes aryl resonances of the product.

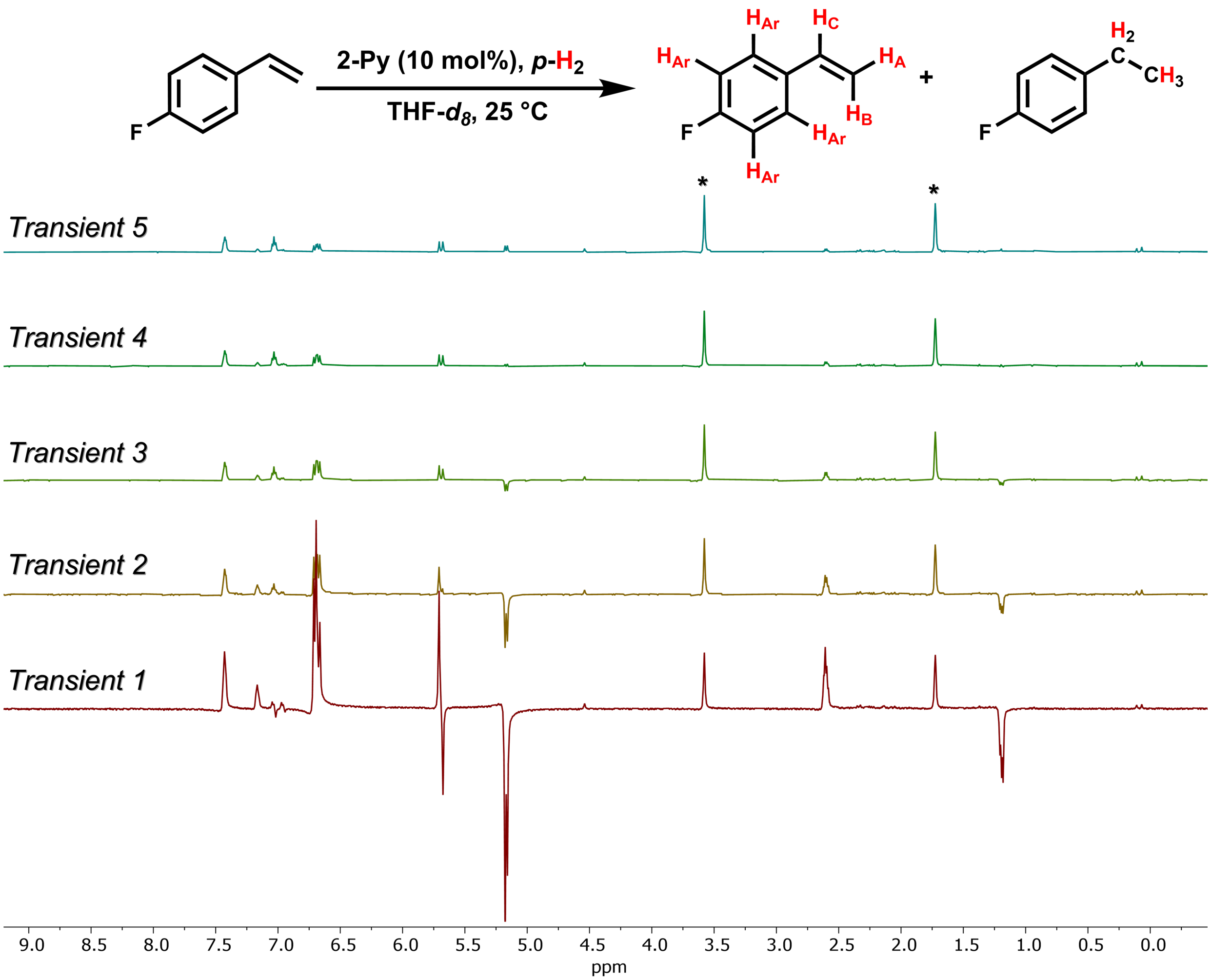

Figure S28 One-transient array of ^{1}H NMR spectra showing the decay of polarization of 4-fluorostyrene in the reaction with *p*-H_2 and **2-Py** at the 50G fringe line of the magnet and with a 45° pulse**.** Bottom spectrum was collected immediately after shaking the sample and inserting into the instrument, followed by an acquisition time of 4.096 s before collection of each subsequent transient. Sample was only shaken once. (*)Denotes THF

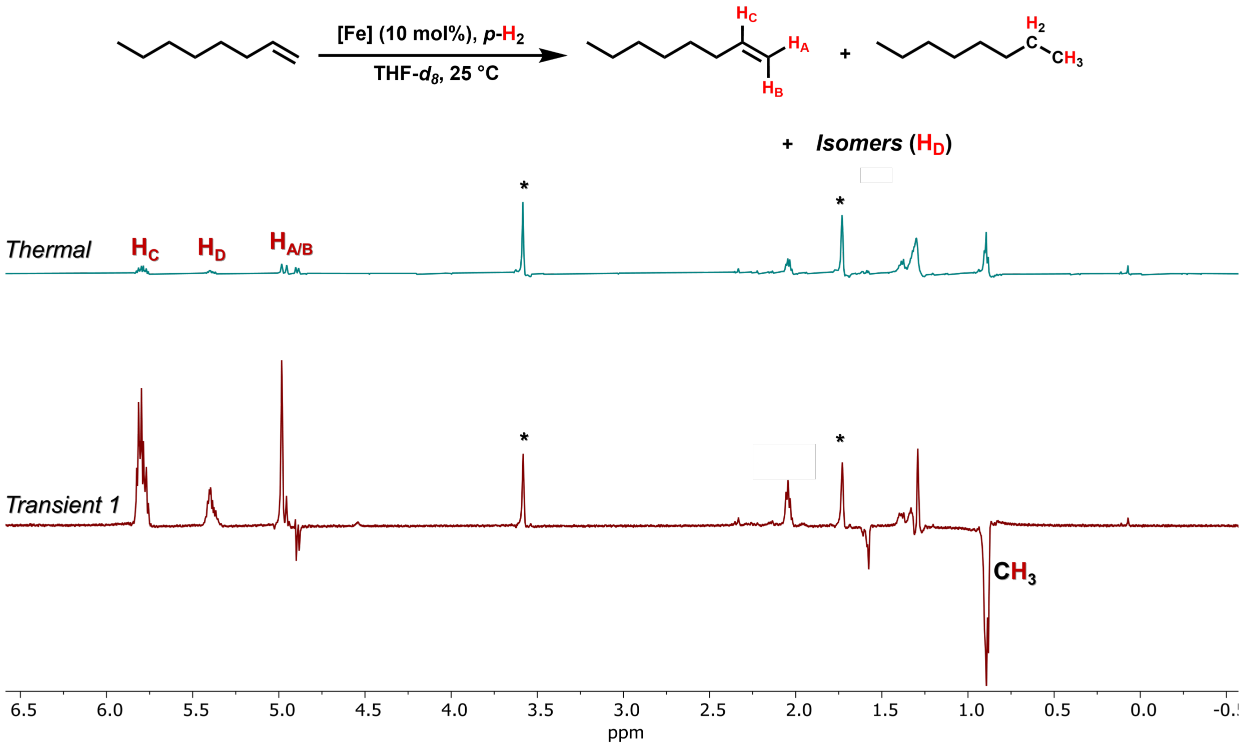

Figure S29 Normalized single transient ^{1}H NMR spectra of the reaction of 1-octene with *p*-H_2 (4 atm) in the presence of **2-Py**. Bottom spectrum was collected immediately following addition of *p*-H_2, while the top spectrum was taken after full relaxation of the nuclear spins back to thermal equilibrium.

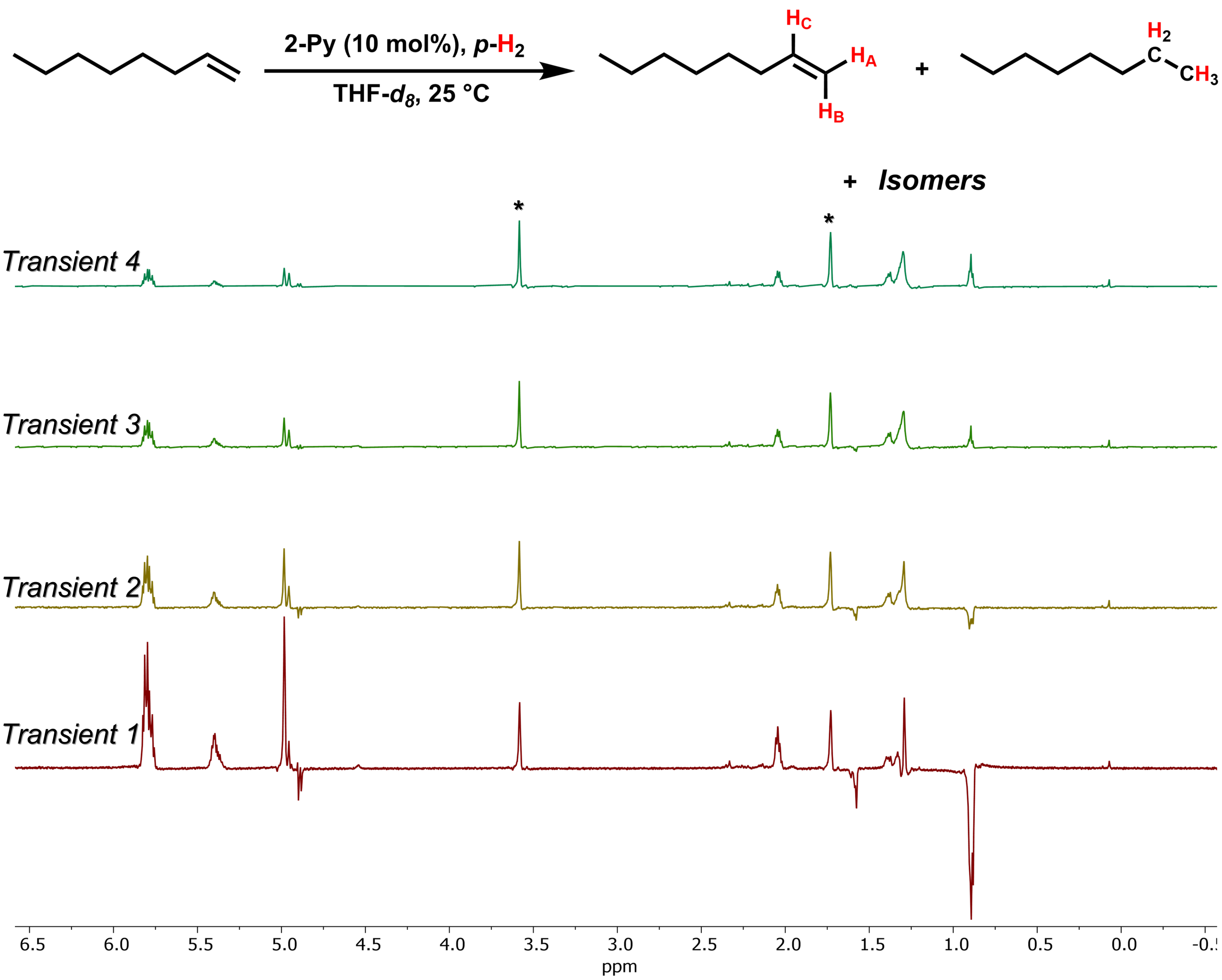

Figure S30 One-transient array of ^{1}H NMR spectra showing the decay of polarization of 1-octene in the reaction with *p*-H_2 and **2-Py** at the 50G fringe line of the magnet and with a 45° pulse**.** Bottom spectrum was collected immediately after shaking the sample and inserting into the instrument, followed by an acquisition time of 4.096 s before collection of each subsequent transient. Sample was only shaken once. (*)Denotes THF

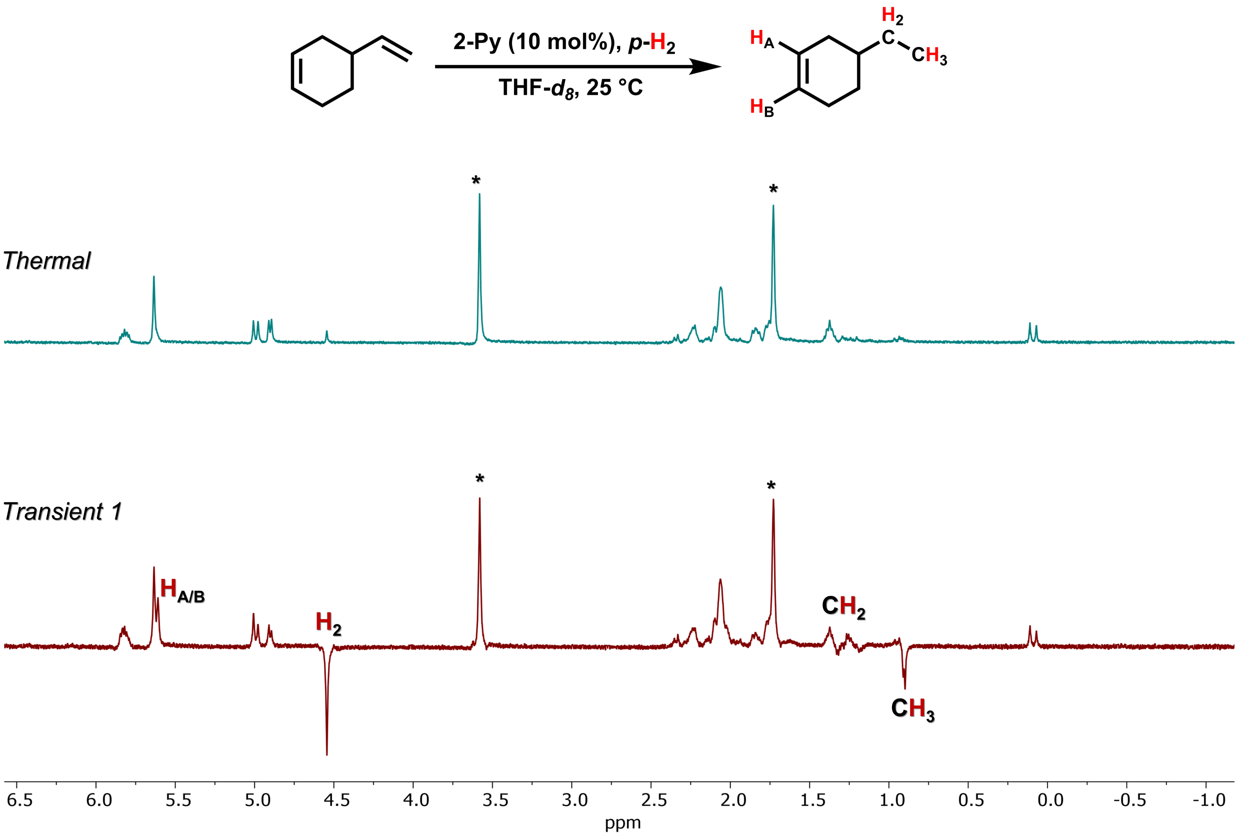

Figure S31 Normalized single transient ^{1}H NMR spectra of the reaction of 4-vinylcyclohexene with p-H_2 (4 atm) in the presence of **2-Py**. Bottom spectrum was collected immediately following addition of p-H_2, while the top spectrum was taken after full relaxation of the nuclear spins back to thermal equilibrium. (^) Denotes olefinic protons of 4-ethylcyclohexene.

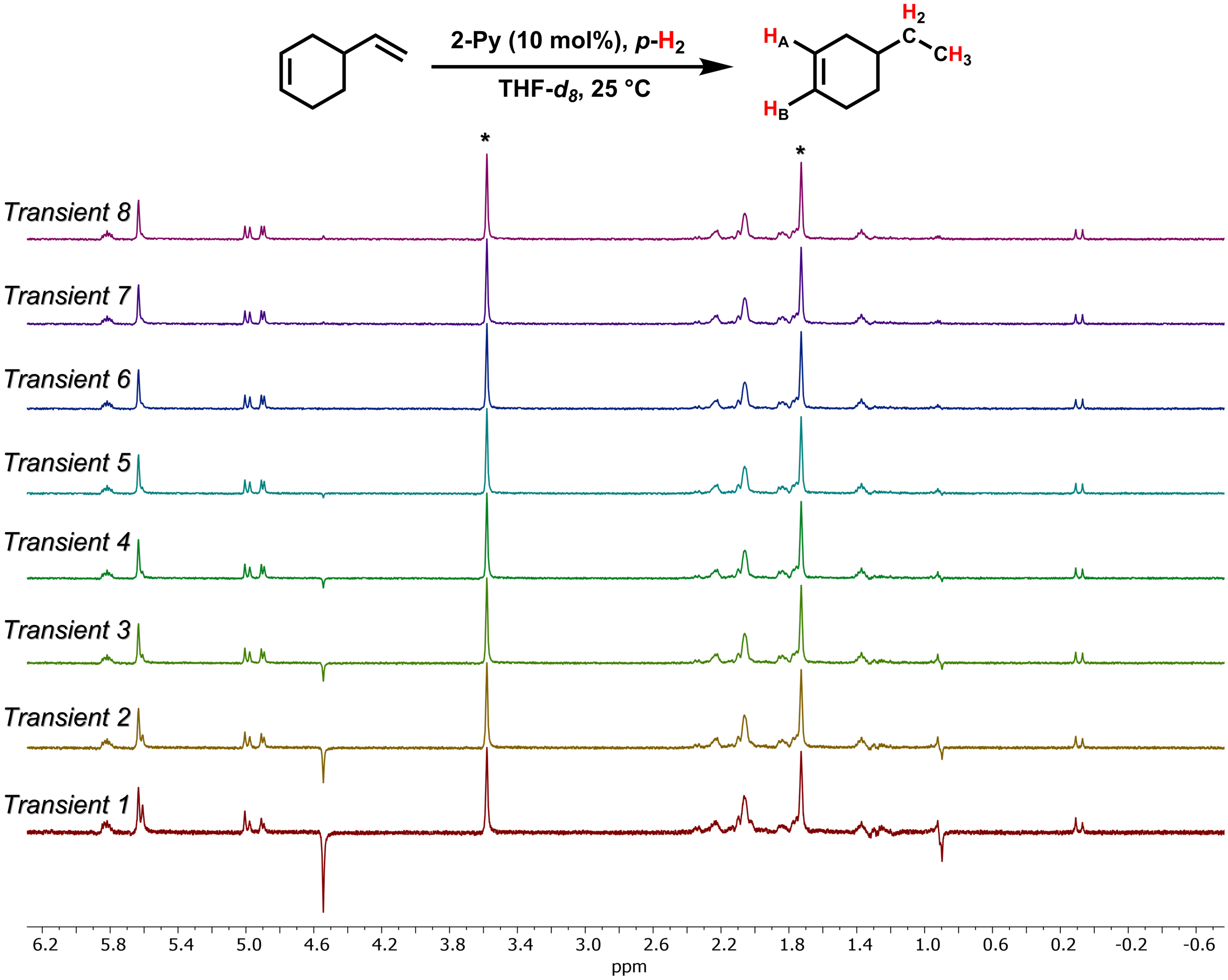

Figure S32 One-transient array of ^{1}H NMR spectra showing the decay of polarization of 4-vinylcyclohexene in the reaction with p-H_2 and **2-Py** at the 50G fringe line of the magnet and with a 45° pulse**.** Bottom spectrum was collected immediately after shaking the sample and inserting into the instrument, followed by an acquisition time of 4.096 s before collection of each subsequent transient. Sample was only shaken once. (*)Denotes THF

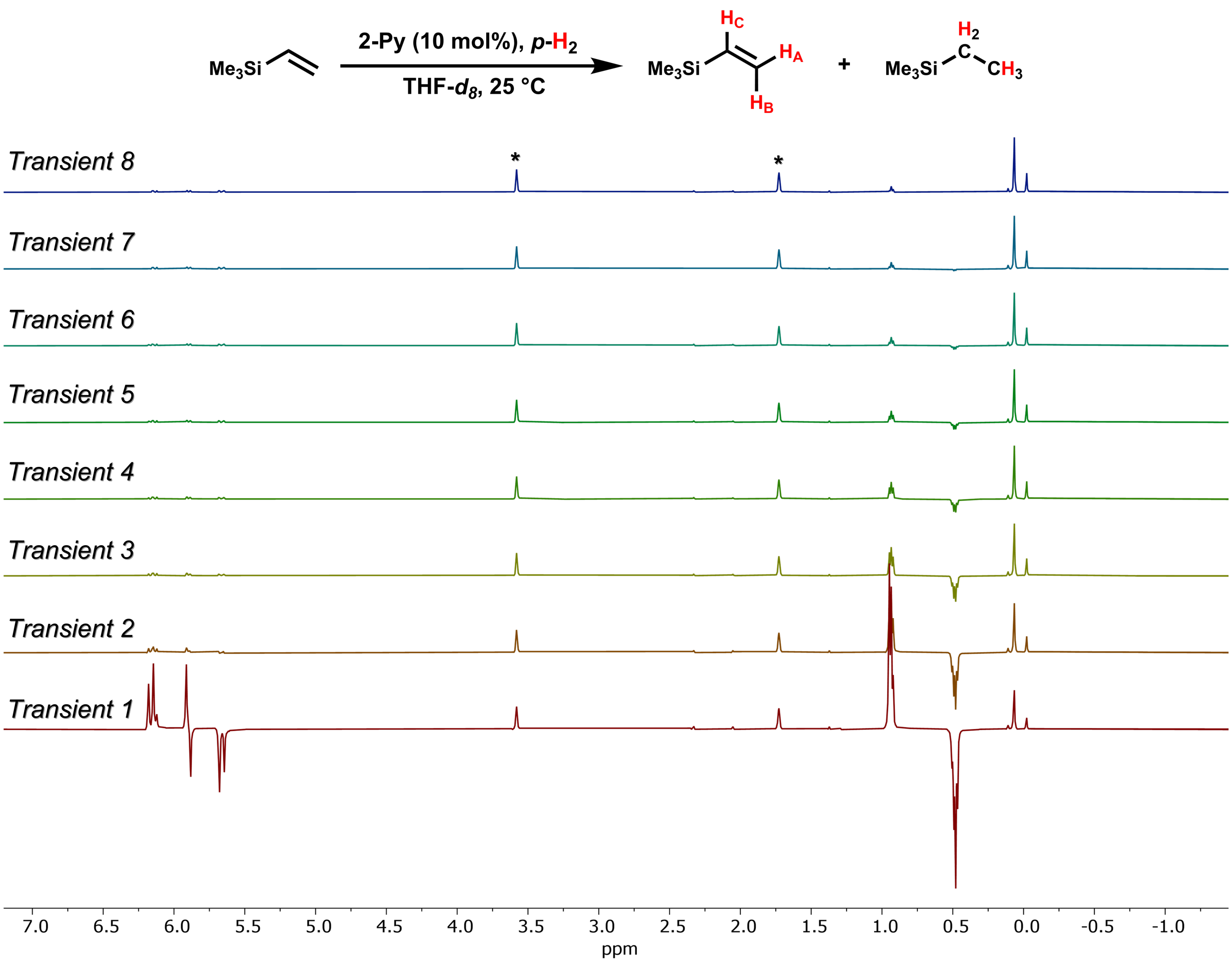

Figure S33 One-transient array of ^{1}H NMR spectra showing the decay of polarization of vinyltrimethylsilane in the reaction with *p*-H_2 and **2-Py** at the 50G fringe line of the magnet and with a 45° pulse**.** Bottom spectrum was collected immediately after shaking the sample and inserting into the instrument, followed by an acquisition time of 4.096 s before collection of each subsequent transient. Sample was only shaken once. (*)Denotes THF

Crystallographic parameters

	**2-Py**	**2-PMe$_3$**	**2-PPh$_3$**
Empirical formula	$C_{86}H_{78}Fe_2N_{12}$	$C_{41}H_{43}FeN_6P$	$C_{56}H_{49}FeN_6P$
Formula weight	1391.30	706.63	892.83
Temperature	100.00 K	100.00 K	100.00 K
Crystal system	Triclinic	Monoclinic	Triclinic
Space group	P-1	P2(1)/n	P-1
Unit Cell Dimensions	a = 13.0198(3) Å<br>b = 15.5970(3) Å<br>c = 15.6396(3) Å<br>α = 60.3051(4)°<br>β = 66.8239(5)°<br>γ = 70.6689(5)°	a = 7.926(3) Å<br>b = 16.642(7) Å<br>c = 27.061(0) Å<br>α = 90°<br>β = 90.368(11)°<br>γ = 90°	a = 12.2862(3) Å<br>b = 13.3264(3) Å<br>c = 16.2887(4) Å<br>α = 72.0170(10)°<br>β = 89.7090(10)°<br>γ = 64.1420(10)°
Volume	2499.13(9) Å^3	3569(2) Å^3	2256.39(10) Å^3
Z	1	4	2
Reflections collected	187324	81429	108785
Independent reflections	12464<br>R_{int} = 0.0417<br>R_{sigma} = 0.0162	6592<br>R_{int} = 0.0530<br>R_{sigma} = 0.0234	11261<br>R_{int} = 0.0392<br>R_{sigma} = 0.0189
Goodness-of-fit on F^2	1.046	1.109	1.044
Final R indexes [I>=2σ (I)]	R_1 = 0.0421<br>wR_2 = 0.1283	R_1 = 0.0479<br>wR_2 = 0.1189	R_1 = 0.0311<br>wR_2 = 0.0793

References

1. A. B. Pangborn, M. A. Giardello, R. H. Grubbs, R. K. Rosen and F. J. Timmers, *Organometallics*, 1996, **15**, 1518–1520.
2. A. D. Ibrahim, K. Tokmic, M. R. Brennan, D. Kim, E. M. Matson, M. J. Nilges, J. A. Bertke and A. R. Fout, *Dalt. Trans.*, 2016, **45**, 9805–9811.
3. G. E. . Martinez, J. A. Killion, B. J. Jackson and A. R. Fout, *Inorg. Synth.,* 2018, **37**, 50–55.
4. APEX2; Bruker AXS, Inc.: Madison, WI, 2004.
5. L. Krause, R. Herbst-Irmer, G. M. Sheldrick and D. Stalke, *J. Appl. Crystallogr*. 2015, **48**, 3−10.
6. G. M. Sheldrick, *Acta Cryst.,* 2015, **A71**, 3−8.
7. G. M. Sheldrick, *Acta Cryst.,* 2015, **C71**, 3−8.
8. O. V. Dolomanov, L. J. Bourhis, R. J. Gildea, J. A. K. Howard and H. Puschmann, *J. Appl. Crystallogr.* 2009, **42**, 339−341.
9. J.-D. Chai and M. Head-Gordon, *Phys. Chem. Chem. Phys.*, 2008, **10**, 6615–6620.
10. Gaussian 09, Revision **E.01**, M. J. Frisch, G. W. Trucks, H. B. Schlegel, G. E. Scuseria, M. A. Robb, J. R. Cheeseman, G. Scalmani, V. Barone, G. A. Petersson, H. Nakatsuji, X. Li, M. Caricato, A. V. Marenich, J. Bloino, B. G. Janesko, R. Gomperts, B. Mennucci, H. P. Hratchian, J. V. Ortiz, A. F. Izmaylov, J. L. Sonnenberg, D. Williams-Young, F. Ding, F. Lipparini, F. Egidi, J. Goings, B. Peng, A. Petrone, T. Henderson, D. Ranasinghe, V. G. Zakrzewski, J. Gao, N. Rega, G. Zheng, W. Liang, M. Hada, M. Ehara, K. Toyota, R. Fukuda, J. Hasegawa, M. Ishida, T. Nakajima, Y. Honda, O. Kitao, H. Nakai, T. Vreven, K. Throssell, J. A., Jr. Montgomery, J. E. Peralta, F. Ogliaro, M. J. Bearpark, J. J. Heyd, E. N. Brothers, K. N. Kudin, V. N. Staroverov, T. A. Keith, R. Kobayashi, J. Normand, K. Raghavachari, A. P. Rendell, J. C. Burant, S. S. Iyengar, J. Tomasi, M. Cossi, J. M. Millam, M. Klene, C. Adamo, R. Cammi, J. W. Ochterski, R. L. Martin, K. Morokuma, O. Farkas, J. B. Foresman, D. J. Fox, Gaussian, Inc., Wallingford CT, 2009.
11. (a) P. J. Hay and W. R. Wadt, *J. Chem. Phys.*, 1985, **82**, 270–283. (b) P. J. Hay and W. R. Wadt, *J. Chem. Phys.*, 1985, **82**, 299–310.
12. A. W. Ehlers, M. Böhme, S. Dapprich, A. Gobbi, A Höllwarth, V. Jonas, K. F. Köhler, R. Stegmann, A. Veldkamp and G. Frenking, *Chem. Phys. Lett.*, 1993, **208**, 111–114.
13. A. V. Marenich, C. J. Cramer and D. G. Truhlar, *J. Phys. Chem. B*, 2009, **113**, 6378–6396.
14. J. R. Cheeseman, G. W. Trucks, T. A. Keith and M. J. Frish, *J. Phys. Chem.*, 1996, **104**, 5497-5509.
15. Note that only one signal for this methyl group was observed in the ^{1}H NMR spectra, due to fast rotation for the NMR timescale at room temperature
16. NBO 7.0. E. D. Glendening, J. K. Badenhoop, A. E. Reed, J. E. Carpenter, J. A. Bohmann, C. M. Morales, P. Karafiloglou, C. R. Landis and F. Weinhold, Theoretical Chemistry Institute, University of Wisconsin, Madison, WI (2018).
17. R. F. W. Bader, Atoms in Molecules: A Quantum Theory; Oxford University Press, 1990.
18. T. Lu and F. Chen, *J. Comput. Chem.*, 2012, **33**, 580–592.
19. A. Otero-de-la-Roza, E. R. Johnson and V. Luaña, *Comput. Phys. Commun.*, 2014, **185**, 1007–1018.
20. Y. J. Park, M. N. Peñas-Defrutos, M. J. Drummond, Z. Gordon, O. R. Kelly, I. J. Garvey, K. L.

Gullett, M. García-Melchor and A. R. Fout, *Inorg. Chem.*, 2022, **61**, 8182–8192.
21. M. Álvarez-Moreno, C. de Graaf, N. López, F. Maseras, J. M. Poblet and C. Bo, *J. Chem. Inf. Model.*, 2015, **55**, 95−103.
22. J. Contreras-García, E. R. Johnson, S. Keinan, R. Chaudret, J.-P. Piquemal, D. N. Beratan and W. Yang, *J. Chem. Theory Comput.*, 2011, **7**, 625−632.
